# Loss of *SETD2* in wild‐type *VHL* clear cell renal cell carcinoma sensitizes cells to STF‐62247 and leads to DNA damage, cell cycle arrest, and cell death characteristic of pyroptosis

**DOI:** 10.1002/1878-0261.13770

**Published:** 2024-11-26

**Authors:** Mathieu Johnson, Sandra Turcotte

**Affiliations:** ^1^ Department of Chemistry and Biochemistry Université de Moncton Canada; ^2^ Atlantic Cancer Research Institute Moncton Canada

**Keywords:** clear cell renal cell carcinoma, PIKfyve, pyroptosis, SETD2, STF‐62247, von Hippel–Lindau

## Abstract

Loss of chromosome 3p and loss of heterogeneity of the von Hippel–Lindau (*VHL*) gene are common characteristics of clear cell renal cell carcinoma (ccRCC). Despite frequent mutations on *VHL*, a fraction of tumors still grows with the expression of wild‐type (WT) *VHL* and evolve into an aggressive subtype. Additionally, mutations on chromatin‐modifying genes, such as the gene coding for the histone methyltransferase SET containing domain 2 (*SETD2*), are essential to ccRCC evolution. We previously identified STF‐62247, a small molecule first discovered as a synthetically lethal molecule for *VHL*‐deficient cells by blocking late stages of autophagy. This study investigated how other commonly mutated genes in ccRCC could impact the response to STF‐62247. We showed that *SETD2* inactivation in ccRCC cells expressing WT‐*VHL* became vulnerable to STF‐62247, as indicated by decreases in cell proliferation and survival. Furthermore, activation of the DNA damage response pathway leads to the loss of M‐phase inducer phosphatase 1 (CDC25A) and cell cycle arrest in S phase. Cleavage of both caspase‐3 and gasdermin E suggests that STF‐62247 eliminates WT‐*VHL* ccRCC cells through pyroptosis specifically when *SETD2* is inactivated.

## Graphical abstract

Vulnerability to the small molecule STF‐62247 is observed in *SETD2*‐inactivated *VHL*‐positive clear cell renal cell carcinoma (ccRCC) cells. DNA damage and loss of CDC25A leads to cell cycle arrest in S phase. Cleavage of caspase‐3 (CASP3) and gasdermin E (GSDME) suggests cell death by pyroptosis.

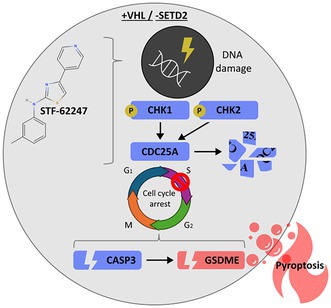

**Abbreviations**
7‐AAD: 7‐aminoactinomycin D
ATG: autophagy‐related protein
ATR: ataxia telangiectasia and Rad3‐related protein
BAP1: BRCA1 associated protein‐1
ccRCC: clear cell RCC
CDC25A: M‐phase inducer phosphatase 1
CDT1: chromatin licensing and DNA replication factor 1
CHK: checkpoint kinase
CRISPR/Cas9: clustered regularly interspaced short palindromic repeats with CRISPR‐associated protein 9
CTL: control
EV: empty vector
gRNA: guide RNA
GSDME: gasdermin E
H3K36me3: Histone H3 trimethylation on lysine 36
HIF: hypoxia‐inducible factor
KIRC: Kidney Renal Clear Cell Carcinoma
PARP: poly(ADP‐ribose) polymerase 1
PBRM1: polybromo 1
PD‐1: programmed cell death protein 1
PI: propidium iodide
PIKfyve: phosphoinositide kinase, FYVE‐type zinc finger containing
RCC: renal cell carcinoma
SETD2: SET containing domain 2
STAT1: signal transducer and activator of transcription 1
TCGA: The Cancer Genome Atlas
TRACERx: TRAcking Cancer Evolution through therapy (Rx)
VEGF: vascular endothelial growth factor
VEGFR: VEGF receptor
VHL: von Hippel–Lindau
WT: wildtype

## 1 Introduction

Renal cell carcinoma (RCC) is the most prevalent type of kidney cancer, with clear cell renal cell carcinoma (ccRCC) being the most common histological subtype [1]. For many years, ccRCCs have been characterized by their well‐known loss of chromosome 3p followed by the inactivation of the tumor suppressor gene von Hippel–Lindau (*VHL*), found on chromosome 3p25.3 [2, 3]. The VHL protein is part of an E3 ubiquitin ligase complex widely studied for its regulation of hypoxia‐inducible factors (HIFs) [4]. In addition to the loss of heterozygosity brought by the alteration of chromosome 3p, the *VHL* gene can be inactivated by hypermethylation of its promoter or by mutations [5, 6]. On the other hand, ccRCCs can occasionally grow with a wild‐type (WT) *VHL* gene, which is characteristic of an aggressive subtype [7]. This was further highlighted by the prospective and longitudinal study TRAcking Cancer Evolution through therapy (Rx) (TRACERx) Renal in which one of the seven ccRCC evolutionary subtypes presented is characterized by the presence of WT‐*VHL* [8]. Mutations of three other tumor suppressor genes, breast cancer gene 1 (BRCA1)‐associated protein‐1 (*BAP1*), polybromo 1 (*PBRM1*) & SET domain containing 2 (*SETD2*), also all found on chromosomes 3p, play important roles as drivers in the evolution of many of these seven subtypes.

SETD2 is canonically known as a histone methyltransferase, catalyzing histone H3 trimethylation on lysine 36 (H3K36me3) [9]. Indeed, many SETD2 functions, such as transcriptional regulation, alternative splicing, DNA repair, and replication, are linked to H3K36me3 [10, 11]. More recently, it has been discovered that SETD2 has H3K36me3‐independent functions, which are related to nonhistone targets such as enhancer of zeste 2 polycomb repressive complex 2 subunit (EZH2), signal transducer and activator of transcription 1 (STAT1), α‐tubulin, actin, and FAK family kinase‐interacting protein of 200 kDa (FIP200) [12, 13, 14, 15, 16]. Importantly, *SETD2* mutations are found in a wide range of cancers, including colorectal and lung cancer [17]. In ccRCC, *SETD2* is mutated in approximately 10–15% of cases, with its loss being associated with a worse prognosis [18, 19].

In the early stages, surgery is the primary curative therapeutic approach used for kidney cancer. However, for advanced RCC, treatment options typically include immunotherapy [anti‐programmed cell death protein 1 (PD‐1) and anti‐cytotoxic T‐lymphocyte‐associated protein 4 (CTLA‐4) antibodies], or targeted therapy [vascular endothelial growth factor (VEGF)/VEGF receptor (VEGFR) or mTOR inhibitors] mostly in combination [20]. Despite advancements in therapeutic approaches, metastatic RCCs frequently resist or develop resistance to these therapies, highlighting the importance of finding new ways to bypass or completely avoid that resistance. With a synthetic lethality screening of 64 000 molecules, our previous work demonstrated the capacity to eliminate *VHL*‐deficient cells with the small molecule STF‐62247 by disrupting the autophagic process [21]. With establishment of new tools to characterize the autophagic flux, we reported that STF‐62247 is blocking late stages of autophagy, specifically lysosome fission, which results in swelling of endolysosomes in *VHL*‐deficient cells. Then, we reclassified STF‐62247 as a blocker of late stages of autophagy [22]. Similarly, enlargement of endolysosomes have been observed and characterized using phosphoinositide kinase, FYVE‐type zinc finger containing (PIKfyve) inhibitors [23, 24]. In fact, PIKfyve inhibition blocks reformation of terminal lysosomes [25]. This can be explained by lysosomal fusion being favored over fission, which causes endolysosomal swelling [26]. We also showed that the loss of *VHL* in ccRCC causes a vulnerability to lysosomal‐disrupting agents [27].

Considering the importance of additional mutations in genes other than *VHL* for the evolution of ccRCC, we wondered how these would influence the response to lysosomal‐disrupting agents, especially using STF‐62247. In this study, we showed that deletion of *SETD2* in ccRCC cells expressing WT‐*VHL* increases the sensitivity to STF‐62247. Under these specific conditions, DNA damage and cell cycle arrest in S phase occur, leading to an annexin V‐positive cell death with cleavage of caspase‐3 and gasdermin E (GSDME), characteristics of pyroptosis.

## 2 Materials and methods

### 2.1 Cell culture and treatments

Both 786‐0 (RRID:CVCL_1051) and 786‐0/VHL cells were a gift from Dr. Amato J. Giaccia (Stanford University, CA, USA), whereas RCC4 (RRID:CVCL_UY81), RCC4/VHL (RRID:CVCL_2706) cells were purchased from Millipore Sigma (Oakville, ON, Canada). HCT116 (RRID:CVCL_0291), A549 (RRID:CVCL_0023), and PC‐3 (RRID:CVCL_0035) cells were purchased from ATCC (Manassas, VA). All cell lines have been authenticated in 2024 by short tandem repeat DNA profiling at Genetica DNA Laboratories (Burlington, NC, USA). 769P (RRID:CVCL_1050), HEK293 (RRID:CVCL_0045) were obtained from ATCC and A704 (RRID:CVCL_1065), RCC‐ER (RRID:CVCL_5870), and Mero‐41 (RRID:CVCL_2592) cells were purchased from Millipore Sigma in the last 3 years. Cells were all incubated in a humidified incubator at 37°C with 5% CO_2_ in their respective media (Table S1). Cells have been tested for mycoplasma contamination. Drugs used for treatments are listed in Table S2.

### 2.2 Western blot

Experiments were performed as previously described [28]. Briefly, cells were lysed in M‐PER lysis buffer containing protease and phosphatase inhibitors. Histones were extracted using the EpiQuik Total Histone Extraction Kit (EpiGentek, Farmingdale, NY, USA). Proteins were separated on SDS/PAGE and transferred on PVDF membranes. Membranes were incubated in blocking solution (5% milk) for 1 h before overnight incubation in primary antibody (List of antibodies in Table S3). The following day, the membranes were washed and incubated in HRP‐conjugated secondary antibody before detection with ECL (Cytiva Amersham, Little Chalfont, Amersham, UK) using a ChemiDoc MP imaging system (BioRad, Hercules, CA, USA).

### 2.3 XTT viability assay

For most cell lines, 5000 cells were plated in each well of a 96‐well plate. Cell lines derived from 786‐0 were plated at 2500 cells per well and 10 000 cells per well for A704. Cells were treated the next day for 72 h. On the last day, media were changed for DMEM high glucose without phenol red containing 0.3 mg·mL^−1^ XTT powder, 2.65 μg·mL^−1^ phenazine methosulfate, and 20% FBS. After incubation at 37°C for 1 h, the absorbance was taken at a wavelength of 450 nm using a SpectraMax i3 (Molecular Devices, San Jose, CA, USA). Viability percentage was calculated with the absorbance ratio between treated and the untreated cells.

### 2.4 CRISPR/Cas9

Guide RNA (gRNA) sequences were designed using Benchling (Sequences listed in Table S4). Following protocol from Shalem et al., sequences were inserted into lentiCRISPRv2 plasmid (blasticidin or hygromycin), gifts from Brett Stringer (Addgene plasmids #98293 and #98291) [29, 30]. Transformation was done in JM109 competent cells. Validation was done by Sanger sequencing. Lentivirus production was performed in HEK293T using the third‐generation system, and cells were transduced by spinfection with 8 μg·mL^−1^ of polybrene.

### 2.5 Gateway cloning

The pENTR™/D‐TOPO™ Cloning Kits (ThermoFisher Scientific, Waltham, MA, USA) was used to prepare the entry vectors for GFP‐SETD2 and HA‐VHL before transformation in TOP10 competent cells. GFP‐SETD2 was amplified from the SETD2‐GFP plasmid (gift from Sérgio De Almeida, Addgene plasmid #80653), and HA‐VHL was amplified from the HA‐VHL wt‐pBabe‐puro plasmid (gift from William Kaelin, Addgene plasmid #19234) [31, 32]. The Gateway™ LR Clonase™ II Enzyme mix was then used to incorporate GFP‐SETD2 into the pLentiCMV/TO Puro DEST (670‐1) and HA‐VHL into the pLentiCMV Blast DEST (706‐1) (gifts from Eric Campeau & Paul Kaufman, Addgene plasmids #17293 & #17451) [33]. Transformation of the novel destination vectors was performed in STBL3 competent cells. Validation of the plasmids was performed by Sanger sequencing. Transfection were performed as previously stated.

### 2.6 Clonogenic assay

Cells were seeded at 300 cells per well in six‐well plates. After 6 h, cells were treated and placed at 37°C with 5% CO_2_. After 7 days, cell colonies were stained with crystal violet and counted. Survival rates were calculated by calculating the ratio of the colonies count from treatment on those from the untreated control.

### 2.7 Cell proliferation and viability

In 12‐well plates, 20 000 cells were plated per well. The next day, cells were treated in duplicate. Starting at Day 0, cells were counted on a hemocytometer using trypan blue. Cell viability represented the number of viable cells relative to the total number of cells (sum of the viable and nonviable cells).

### 2.8 Cell cycle analysis

After 48 h of treatment, cells were trypsinized and for each sample, 1 million cells were collected and centrifuged at 200 ***g*** for 5 min followed by one PBS wash. The pellet was suspended in 300 μL of cold PBS. Cells were then fixed by adding 700 μL of cold ethanol and by inverting tubes to mix. Fixed cells were centrifuged at 300 ***g*** for 5 min. Supernatant was discarded, and cells were washed twice in PBS followed by a centrifugation at 500 ***g*** for 5 min. Pellets were then suspended in 500 μL of FxCycleTM PI/RNase Staining Solution (ThermoFisher Scientific) and incubated in the dark for 15 min prior to samples processing with a CytoFLEX (Beckman Coulter, CA, USA). Analysis was performed using the Kaluza Analysis Software (Beckman Coulter, Brea, IN, USA).

### 2.9 DNA damage immunofluorescence

In a 12‐well plate, 25 000 cells were seeded on coverslips. The next day, cells were treated with 3 μm STF‐62247 for 48 h. Doxorubicin, 1 μm for 4 h, was used as a positive control. Cells were fixed in 3.7% paraformaldehyde for 20 min, permeabilized in 0.25% Triton X‐100 for 5 min, and incubated in 5% FBS blocking solution for 15 min. Then, cells were incubated in γH2AX antibody for 1 h, washed twice in PBS, and incubated for another hour in Alexa fluor secondary antibody. After three PBS washes, 5‐min incubation in DAPI and two more washes, coverslips with stained cells were mounted on slides. Images were acquired by confocal microscopy using an Olympus Fluoview FV1000 (Olympus, Breinigsville, PA, USA).

### 2.10 Annexin V/7AAD

After 48 h of treatment, cells were trypsinized and collected with their respective media. Cells were centrifuged at 200 ***g*** for 5 min before being stained with the FITC Annexin V Apoptosis Detection Kit with 7‐AAD (SKU 35‐6410‐KIT) as per the manufacturer's protocol. Samples were processed on the Attune NxT flow cytometer (Beckman Coulter, CA), and data were analyzed on the Attune^tm^ software.

### 2.11 Statistical analysis

All experiments were performed with a minimum of three biological replicates. Where appropriate, results are presented as the mean and the standard error of the mean (SEM). Statistical analyses were performed with (graphpad prism 9.5.1, Boston, MA, USA).

## 3 Results

### 3.1 ccRCC cell lines with mutations in chromatin remodeling genes respond differently to STF‐62247 and PIKfyve inhibitors

**Fig. 1:**
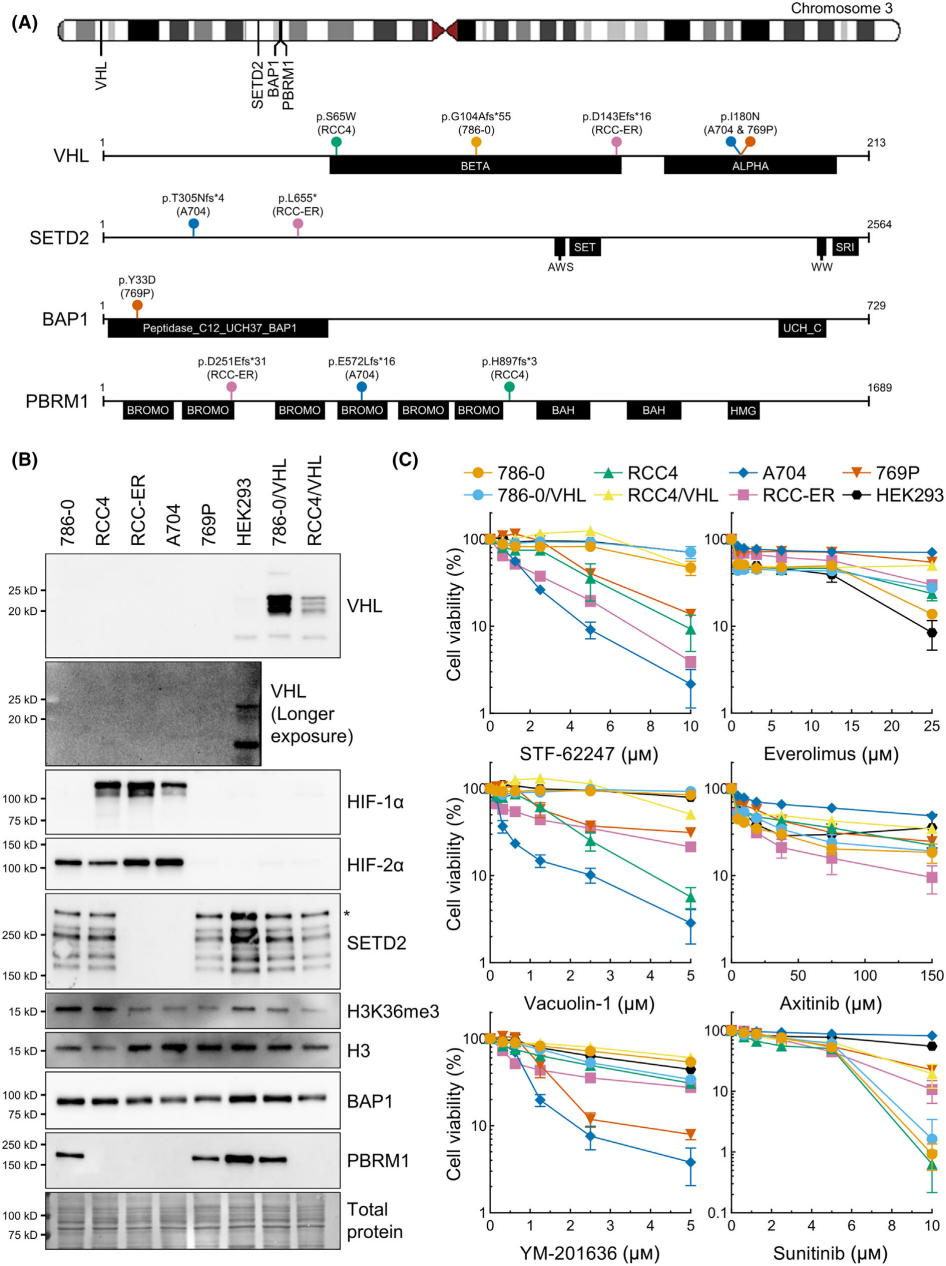
Common mutated genes on chromosome 3p in ccRCC cell lines and sensitivity to diverse agents. (A) Schematic representation of mutations affecting proteins encoded on chromosome 3p in ccRCC cell lines. Mutations indicated were documented on the DepMap portal or in the literature [34, 35, 36]. Protein domains were predicted by the InterPro database [78]. (B) Western blot analysis of commonly altered proteins (VHL, BAP1, PBRM1, SETD2) in ccRCCs was used to demonstrate how mutations impacted protein expression in ccRCC cell lines. Expression of VHL is inversely correlated with HIF‐1α and/or HIF‐2α expression. *Represents SETD2 full‐length of the protein. Linked to SETD2, the detection of H3K36me3 was added with total H3. Total protein stain serves as loading control (*N* = 3). (C) Cell viability measured by XTT assay. Cells were treated with STF‐62247, PIKfyve inhibitors (Vacuolin‐1 and YM‐201636) and targeted therapy agents for ccRCC (Everolimus, Axitinib and Sunitib) for 72 h. Results are presented as the mean ± SEM (*N* = 3). IC50s were determined by nonlinear regression using graphpad prism 9.5.1 (reported in Table 1). ALPHA, alpha domain; AWS, associated with set domain; BAH, bromo adjacent homology domain; BETA, beta domain; BROMO, bromodomain; HMG, high mobility group box domain; SET, Su(var)3–9, enhancer of zeste, and trithorax domain; SRI, Set2 Rpb1 interacting domain; UCH, ubiquitin carboxyl‐terminal hydrolase; WW, WW domain.

To evaluate how ccRCC subtypes respond to STF‐62247 and various treatments, we used cell lines with different mutation patterns on chromosome 3p (Fig. 1A). All the parental ccRCC cell lines are mutated on *VHL*. Mutations for all cell lines were found on the DepMap portal (https://depmap.org/portal), except for RCC4 that have a missense mutation on *VHL* (p.S65W) and a frameshift mutation on *PBRM1* (p.H897fs*3) [34, 35, 36]. Other than BAP1 that is still highly expressed in the 769P cells, all these mutations altered the protein expression of the corresponding genes (Fig. 1B). In addition, histones were extracted from parental cell lines, and immunoblots showed reduction in H3K36me3 expression in *SETD2* mutated cells. The residual expression of H3K36me3 in these cells could be explained by SMYD5 and SETD5 that were shown to be able to catalyze H3K36me3 [37, 38]. It was also reported that *HIF‐1α* mRNA is truncated in 786‐0, preventing its transcription, and that it is also not expressed in 769P, demonstrated by reverse transcription polymerase chain reaction (RT‐PCR) [34].

To assess cytotoxicity of STF‐62247 and a variety of drugs, cells were treated with tyrosine kinase inhibitors, mTOR inhibitor, all related to ccRCC therapies, as well as with autophagy inhibitors, and lysosomal and/or endosomal disrupting agents (Fig. 1C, Table 1, Fig. S1). We also added HEK293 cells, which does not have mutations on any of the genes of interest, and 786‐0 and RCC4 in which *VHL* was reintroduced, respectively, annotated as 786‐0/VHL and RCC4/VHL. As expected, *VHL*‐inactivated cells showed greater sensitivity to STF‐62247 than genetically matched cells expressing WT‐*VHL*. This was also observed in vacuolin‐1 RCC4‐treated cells, a cell‐permeable inhibitor of lysosomal exocytosis also known as PIKfyve inhibitor. Notably, 786‐0 tended to be more resistant to STF‐62247 and PIKfyve inhibitors than the other ccRCC cell lines. Coincidentally, this is the only ccRCC cell line without mutations on *PBRM1*, *SETD2*, or *BAP1*. One striking result was from RCC‐ER and A704 cells, both with mutations on *VHL*, *PBRM1*, and *SETD2*, which had the lowest IC50 values for STF‐62247 (1.268 and 1.393 μm, respectively). This was also true for both PIKfyve inhibitors where the IC50s were under 1 μm. By comparing RCC4 and RCC4/VHL, it was apparent that *VHL* expression increased resistance to drugs targeting autophagy and lysosomes. Altogether, we reiterated that STF‐62247 targets *VHL*‐deficient cells, and we found that cells with additional mutations on *PBRM1* and *SETD2* showed higher sensitivity to STF‐62247 and PIKfyve inhibitors.

**Table 1:** Drugs of interest in kidney cancer and their IC50 values measured in ccRCC cell lines.

		IC50							
		95% confidence interval							
		786‐0	786‐0/VHL	RCC4	RCC4/VHL	RCC‐ER	A704	769P	HEK293
Tyrosine kinase inhibitors	Axitinib (μm)	4.215	12.85	16.52	NA	6.8	149.6	24.88	5.125
		1.999–8.889	8.904–18.54	11.83–23.06	NA	4.240–10.91	92.90–240.8	17.42–35.52	0.2703–97.18
	BAY 43–9006 (μm)	6.62	6.896	16.05	14.43	6.449	11.32	8.383	6.599
		6.157–7.116	6.355–7.482	14.73–17.49	11.80–17.65	5.969–6.968	8.746–14.65	7.402–9.493	5.233–8.322
	Sunitinib (μm)	4.518	4.821	2.935	5.192	4.092	63.79	5.163	15.87
		3.798–5.375	3.781–6.146	2.029–4.247	3.793–7.107	3.614–4.633	7.628–533.5	4.691–5.682	7.932–31.75
mTOR inhibitor	Everolimus (μm)	7.49	6.334	7.027	8.646	11.59	23.68	19.33	6.382
		5.130–10.93	3.844–10.44	4.642–10.64	4.115–18.17	8.942–15.01	15.86–35.36	13.91–26.84	4.369–9.324
Autophagy inhibitor	Bafilomycine A1 (nm)	64.48	106.6	~ 24.13	NA	13.94	60.74	6.087	9.82
		22.16–187.7	23.80–477.8	Very wide	NA	10.73–18.10	20.89–176.6	5.028–7.370	8.407–11.47
	Hydroxychloroquine (μm)	21.5	21.12	34.07	62.59	44.96	39.58	36.9	52.83
		20.71–22.31	18.51–24.11	28.42 to 40.84	47.44–82.57	41.42–48.80	29.02–53.97	31.17–43.70	47.91–58.25
Lysosomal iand/or iendosomal idisrupting iagents	Dynasore (μm)	33.75	49.72	71.78	92.56	40.77	302.9	31.63	19.4
		28.24–40.35	45.06–54.85	61.70–83.49	77.58–110.4	34.42–48.28	224.9–407.9	26.02–38.45	11.40–33.03
	Brefeldin A (nm)	34.8	33.73	55.42	101.1	50.73	79.01	77.23	~ 111.9
		30.90–39.18	30.96–36.75	46.39–66.21	84.77–120.7	46.27–55.62	74.86–83.39	72.59–82.17	Very wide
	CA‐074 methyl ester (μm)	14.89	23.87	20.52	~ 39.98	23.72	NA	~ 41.74	7.334
		12.63–17.55	21.74–26.22	17.17–24.53	Very wide	21.19–26.55	NA	Very wide	6.748–7.970
	STF‐62247 (μm)	12.79	54.25	3.396	~ 9.968	1.268	1.393	4.671	15.5
		6.393–25.60	4.796–613.7	2.412–4.782	Very wide	1.095–1.468	1.337–1.452	3.995–5.461	8.643–27.78
	Vacuolin‐1 (μm)	18.6	NA	1.539	5.707	0.8641	0.1689	2.258	16.46
		12.32–28.08	NA	1.367–1.732	4.120–7.906	0.7581–0.9849	0.09562–0.2985	1.519–3.359	10.31–26.26
	YM‐201636 (μm)	6.12	3.05	2.276	8.308	0.7377	0.8165	1.287	4.469
		5.298–7.069	2.747–3.386	2.080–2.490	7.181–9.612	0.5919–0.9194	0.7601–0.8772	1.103–1.501	2.963–6.741

### 3.2 Inactivation of *SETD2* sensitizes WT‐ *VHL* cells to STF‐62247 and PIKfyve inhibitors

**Fig. 2:**
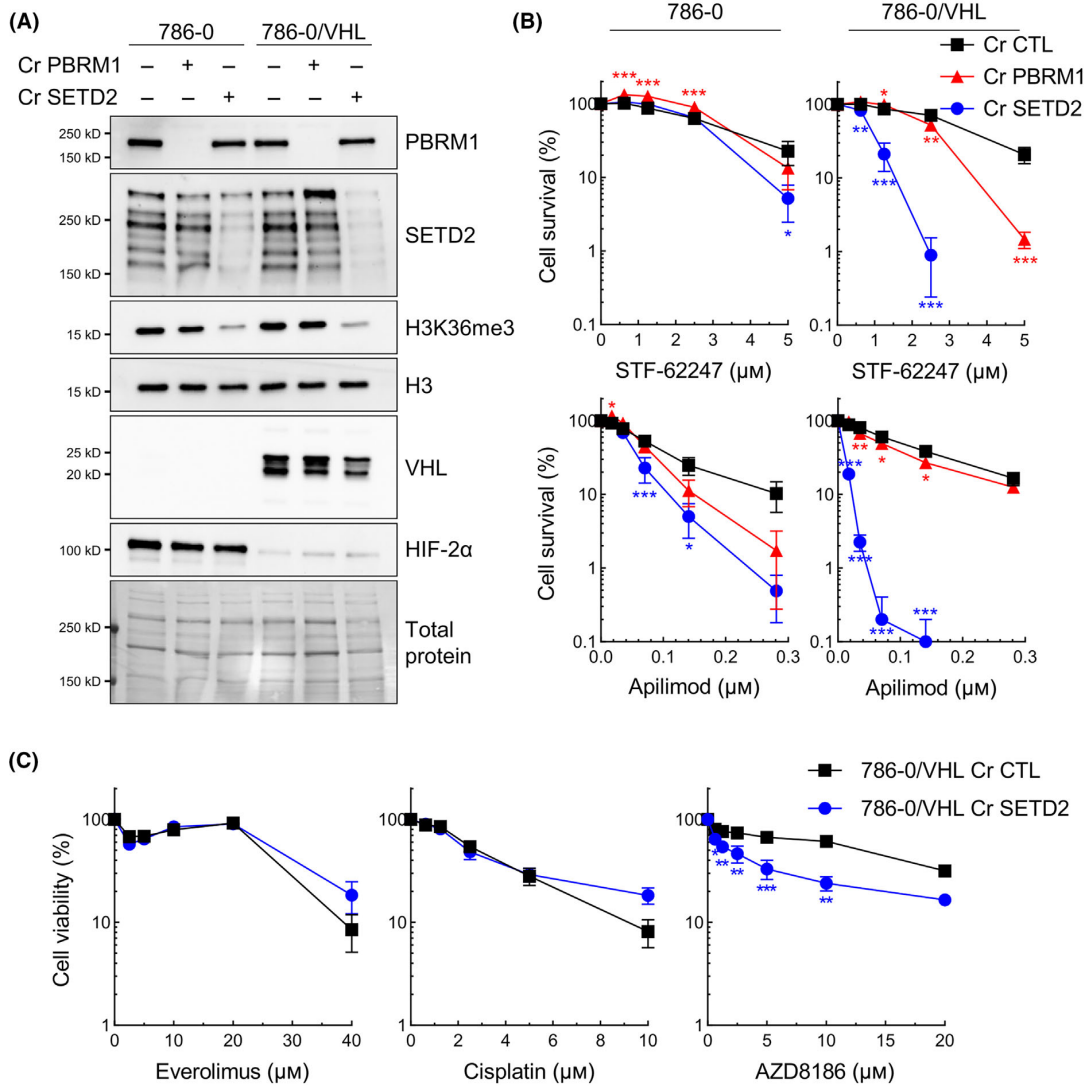
Inactivation of *SETD2* sensitizes cells to STF‐62247 and PIKfyve inhibitors. (A) Western blot analysis validated gene repression by CRISPR/Cas9 of *PBRM1* and *SETD2* (respectively Cr PBRM1 and Cr SETD2) in 786‐0 and 786‐0/VHL cells. Decrease in SETD2 protein expression is accompanied by a decrease in H3K36me3 (*N* = 3). (B) Cell survival was measured by clonogenic assay. *PBRM1*‐ or *SETD2*‐knockout in 786‐0 and 786‐0/VHL were treated with STF‐62447 or apilimod for 7 days before being stained with crystal violet. Control cells with an empty vector are annotated Cr CTL. Cell colonies were counted, and survival was calculated by comparing with an untreated control. Statistical significance was measured using two‐way ANOVA followed by Dunnett's multiple comparisons test to compare Cr PBRM1 or Cr SETD2 with Cr CTL. (C) XTT assay in 786‐0/VHL Cr CTL and 786‐0/VHL Cr1 SETD2 cells exposed to drugs other than STF‐62247 and PIKfyve inhibitors. Cells were treated for 72 h. Statistically significant differences between Cr CTL and Cr SETD2 were tested with two‐way ANOVA followed by Sidak's multiple comparisons test. Results are presented as the mean ± SEM (*N* = 3, *P < 0.05, **P < 0.01, ***P < 0.001).

To better understand the individual impact of PBRM1 and SETD2 in response to STF‐62247 and PIKfyve inhibitors in ccRCC, we used clustered regularly interspaced short palindromic repeats with CRISPR‐associated protein 9 (CRISPR/Cas9) to repress their gene expression in 786‐0 and 786‐0/VHL cells. Indeed, both proteins were decreased by their respective gRNA in addition to a decrease in H3K36me3 levels in Cr SETD2 (annotated Cr1 SETD2 from this point on) (Fig. 2A). Inactivation of *PBRM1* in both 786‐0 and 786‐0/VHL modestly influenced the response to STF‐62247 and apilimod, a PIKfyve inhibitor used in clinical trials for non‐Hodgkin lymphoma (NCT02594384) (Fig. 2B). Surprisingly, 786‐0/VHL Cr1 SETD2 showed a significant sensitivity to both treatments. For STF‐62247 specifically, IC50 went from 4.024 to 0.908 μm after inactivation of *SETD2* in 786‐0/VHL, which was even lower than in 786‐0 Cr control (CTL) that had an IC50 of 3.503 or 2.882 μm in 786‐0 Cr SETD2. These results were confirmed in a second clone of 786‐0/VHL cells with a different gRNA targeting *SETD2* (annotated Cr3 SETD2) (Fig. S2A,B). We also stably repressed *SETD2* expression in RCC4 and RCC4/VHL, and by measuring cell viability in heterogeneous cell pools, *VHL*‐positive cells were still more affected by PIKfyve inhibitors (Fig. S3A,B).

To determine whether this sensitivity was specific to STF‐62247 and PIKfyve inhibitors and not just to a general vulnerability to any stresses, we measured cell viability with other agents. We used everolimus, cisplatin for which *SETD2*‐deficient cells in lung cancer showed more resistance [39], and AZD8186, a phosphoinositide 3‐kinases (PI3K)β/δ inhibitor, which has been shown to target *SETD2*‐deficient cells in ccRCC [40]. There was no significant difference in 786‐0/VHL Cr CTL cytotoxicity using everolimus or cisplatin, but as expected, *SETD2*‐deficient cells were more sensitive to AZD8186 (Fig. 2C). These results indicated that the loss of *SETD2* can confer vulnerability to specific drugs.

### 3.3 Vulnerability to STF‐62247 and PIKfyve inhibitors is specific to ccRCC

**Fig. 3:**
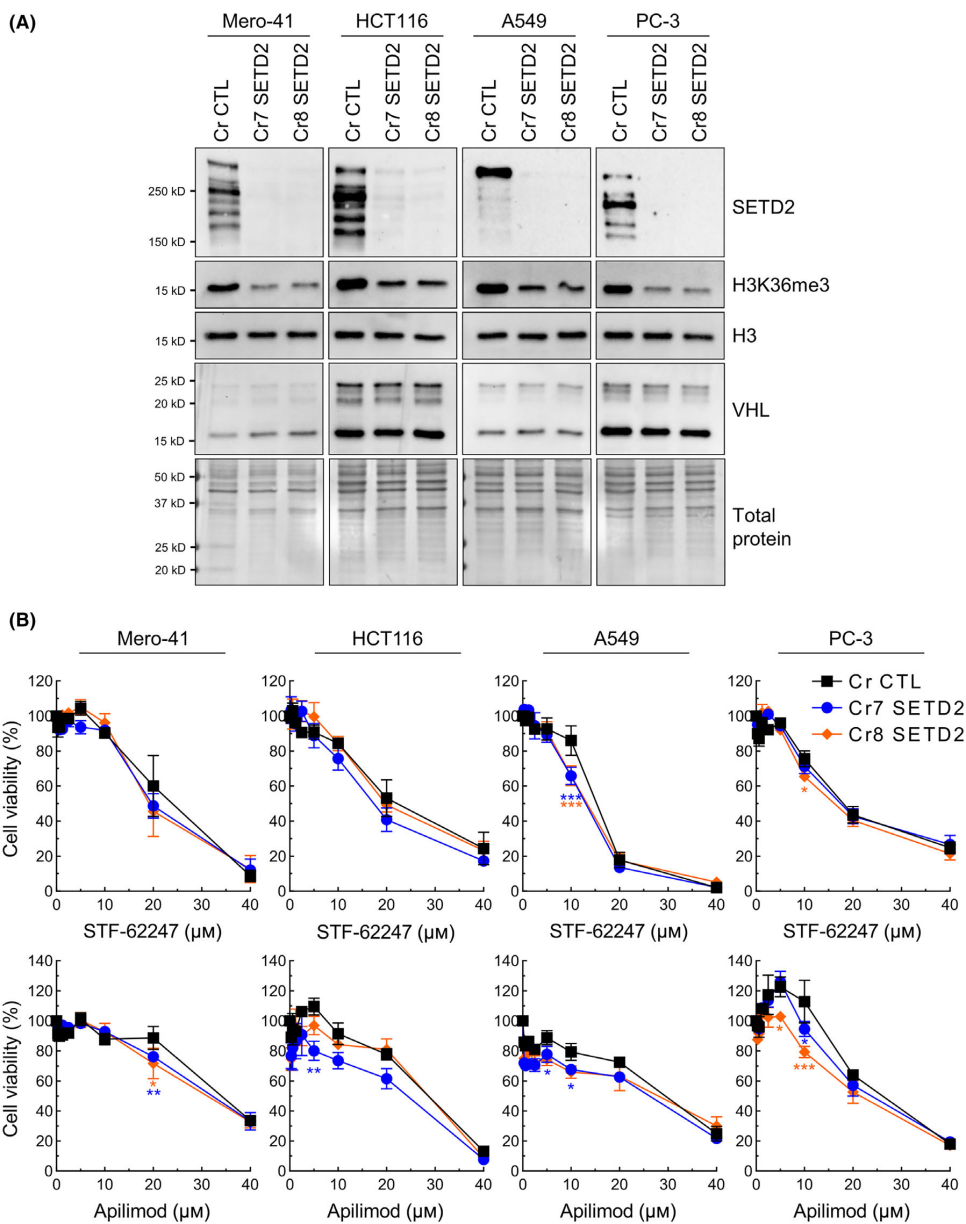
Sensitivity to STF‐62247 and PIKfyve inhibitors is specific to ccRCC. (A) Western blot analysis validated *SETD2*‐knockout following CRISPR/Cas9 gene repression in Mero‐41 (mesothelioma), HCT116 (colon cancer), A549 (lung cancer) and PC‐3 (prostate cancer) cells. Control cells with an empty vector are annotated Cr CTL. Two different gRNA targeting *SETD2* were used (annotated Cr7 and Cr8). Decrease in SETD2 protein expression is accompanied by a decrease in H3K36me3 (*N* = 3). (B) Cell viability was measured by XTT assay. Cells were treated with STF‐62247 or the PIKfyve inhibitor apilimod for 72 h. Statistical significance was measured using two‐way ANOVA followed by Dunnett's multiple comparisons test to compare Cr7 SETD2 or Cr8 SETD2 with Cr CTL. Results are presented as the mean ± SEM (*N* = 3, *P < 0.05, **P < 0.01, ***P < 0.001).

Since we observed cytotoxicity in response to STF‐62247 or apilimod in *SETD2*‐knockout cells expressing VHL, we decided to inactivate *SETD2* in cell lines from other cancers known to have mutations on *SETD2*, but not on *VHL*. Thus, we used Mero‐41, HCT116, A549, and PC‐3 cell lines, respectively, from mesothelioma, colon, lung, and prostate cancer. Again, we used CRISPR/Cas9 to repress *SETD2* gene expression with two different gRNA. An impressive decrease in SETD2 and H3K36me3 protein levels was obtained with both sequences in all cell lines (Fig. 3A). We used XTT viability assay to measure their response to STF‐62247, apilimod, vacuolin‐1, and APY0201. Interestingly, the four cancer cell lines were mostly unaffected by PIKfyve inhibitors when *SETD2* was inactivated (Fig. 3B, Fig. S4). Moreover, compared with ccRCC cells, these parental cells derived from cancer outside the kidney exhibit greater resistance to STF‐62247 and PIKfyve inhibitors, which suggest that ccRCCs are more dependent on PIKfyve activity and associated cellular processes, supporting our previous work [27].

### 3.4 STF‐62247 causes a decrease in cell proliferation and viability

**Fig. 4:**
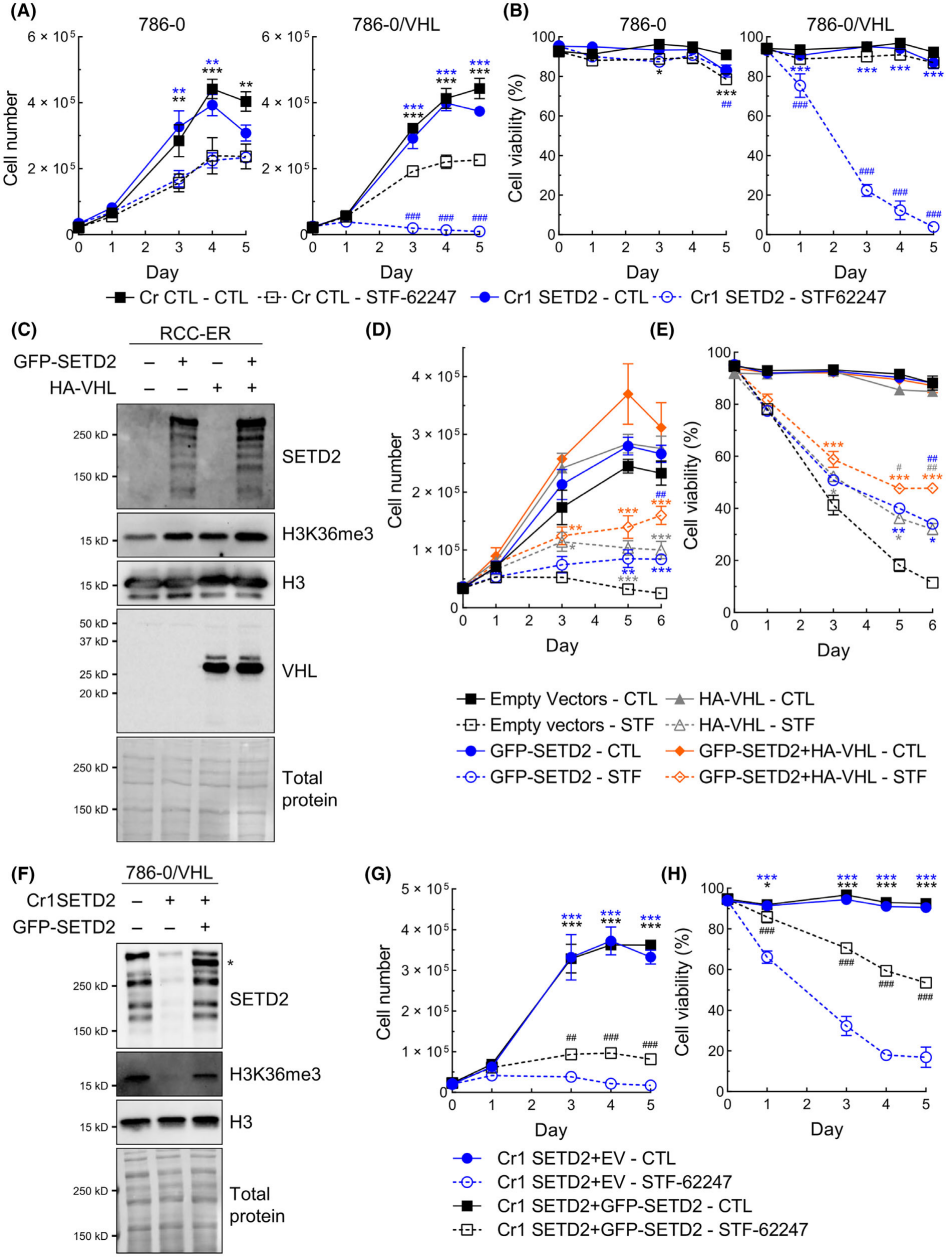
STF‐62247 decreases cell proliferation and viability of *SETD2*‐inactivated cells expressing WT‐*VHL*. (A) Proliferation and (B) viability were measured by counting 786‐0 and 786‐0/VHL cells (Cr CTL and Cr1 SETD2) with trypan blue. Cells were treated with 3 μm STF‐62247 on day 0. Results are presented as the mean ± SEM (*N* = 3). Statistically significant differences were tested with a two‐way ANOVA followed by Tukey's multiple comparisons test. Comparisons between CTL and STF‐62247 are indicated with the * symbol and comparisons between Cr1 SETD2 and Cr CTL are indicated with the # symbol. (C) Western blot of SETD2, H3K36me3, H3 and VHL in RCC‐ER expressing GFP‐SETD2 and/or HA‐VHL. Empty vectors (670‐1 vector for GFP‐SETD2 and 706‐1 vector for HA‐VHL) were used when the respective gene was not reintroduced (*N* = 3). (D) Proliferation and (E) viability were measured by counting cells with trypan blue. RCC‐ER cells were treated with 1.25 μm STF‐62247 on day 0. Results are presented as the mean ± SEM (*N* = 3). Statistically significant differences were tested with a two‐way ANOVA followed by Tukey's multiple comparisons test. Comparisons of GFP‐SETD2, HA‐VHL or GFP‐SETD2 + HA‐VHL with the Empty Vectors (only with STF‐62247) are indicated with the * symbol. Comparisons between GFP‐SETD2 or HA‐VHL with GFP‐SETD2 + HA‐VHL are indicated with the # symbol. No significant differences were observed between GFP‐SETD2 and HA‐VHL. (F) Validation, by western blot, of GFP‐SETD2 reintroduction in 786‐0/VHL Cr1 SETD2 (compared to 786‐0/VHL Cr1 SETD2 with the empty vector (Cr1 SETD2^+^, GFP‐SETD2^−^). 786‐0/VHL Cr CTL, in the first lane, was used as a comparison. GFP‐SETD2 is indicated by an asterisk (*) (*N* = 3). (G) Proliferation and (H) viability were measured by counting cells with trypan blue. Cells were treated with 3 μm STF‐62247 on day 0. Results are presented as the mean ± SEM (*N* = 3). Statistically significant differences were tested with a two‐way ANOVA followed by Tukey's multiple comparisons test. Comparisons between CTL and STF‐62247 are indicated with the * symbol and comparisons between 786‐0/VHL Cr1 SETD2 EV and GFP‐SETD2 are indicated with the # symbol (*P < 0.05, **P < 0.01, ***P < 0.001 or ^#^ *P* < 0.05, ^##^ *P* < 0.01, ^###^ *P* < 0.001).

We further investigated whether the sensitivity of WT‐*VHL* ccRCC cells to STF‐62247 after *SETD2* inactivation was associated with a decrease in proliferation or with cell death. In both 786‐0 and 786‐0/VHL, *SETD2* inactivation alone had no significant effect on cell proliferation or viability. When treated with STF‐62247, we observed a decrease in proliferation in all cell lines, but proliferation was completely abolished only in both clones of 786‐0/VHL Cr SETD2 (Fig. 4A,B, Fig. S5A,B). Additionally, the viability of 786‐0/VHL Cr1 SETD2 cells significantly dropped to reach 22.3% after 3 days. To confirm these results, clones were selected from previously used RCC4/VHL Cr2 and Cr3 SETD2, and cell counts were performed similarly. Likewise, in both clones, the inactivation of *SETD2* made RCC4/VHL vulnerable to STF‐62247, as shown by decreases in cell viability and proliferation (Fig. S6A–C).

As a complementary approach, we decided to reintroduce *SETD2* in addition to HA‐VHL in the RCC‐ER cell line deficient in both genes. As others before us, we were unable to reintroduce full‐length *SETD2*, and we have decided to reintroduce a GFP‐tagged truncated version of *SETD2* (missing the first 503 residues in the N terminus but still containing all the functional domains) (Fig. 4C) [41]. The reintroduction of both genes together helped the cells resist STF‐62247 (Fig. 4D,E). Restoration of both genes alone also confers, to a lesser extent, resistance to STF‐62247. We were also able to do the reintroduce GFP‐SETD2 in the 786‐0/VHL Cr1 SETD2 (Fig. 4F). Notably, in addition to the expression of the exogenous GFP‐SETD2, an increase in the highest band, allegedly endogenous full‐length SETD2, was observed after we reintroduced GFP‐SETD2. Moreover, reintroduction of GFP‐SETD2 partially rescued proliferation and viability in response to STF‐62247, whereas 70.5% of the cells still viable after 3 days compared with 32.3% from the 786‐0/VHL Cr1 SETD2 (Fig. 4G,H).

### 3.5 S phase arrest in *SETD2* ‐deficient cells treated with STF‐62247

**Fig. 5:**
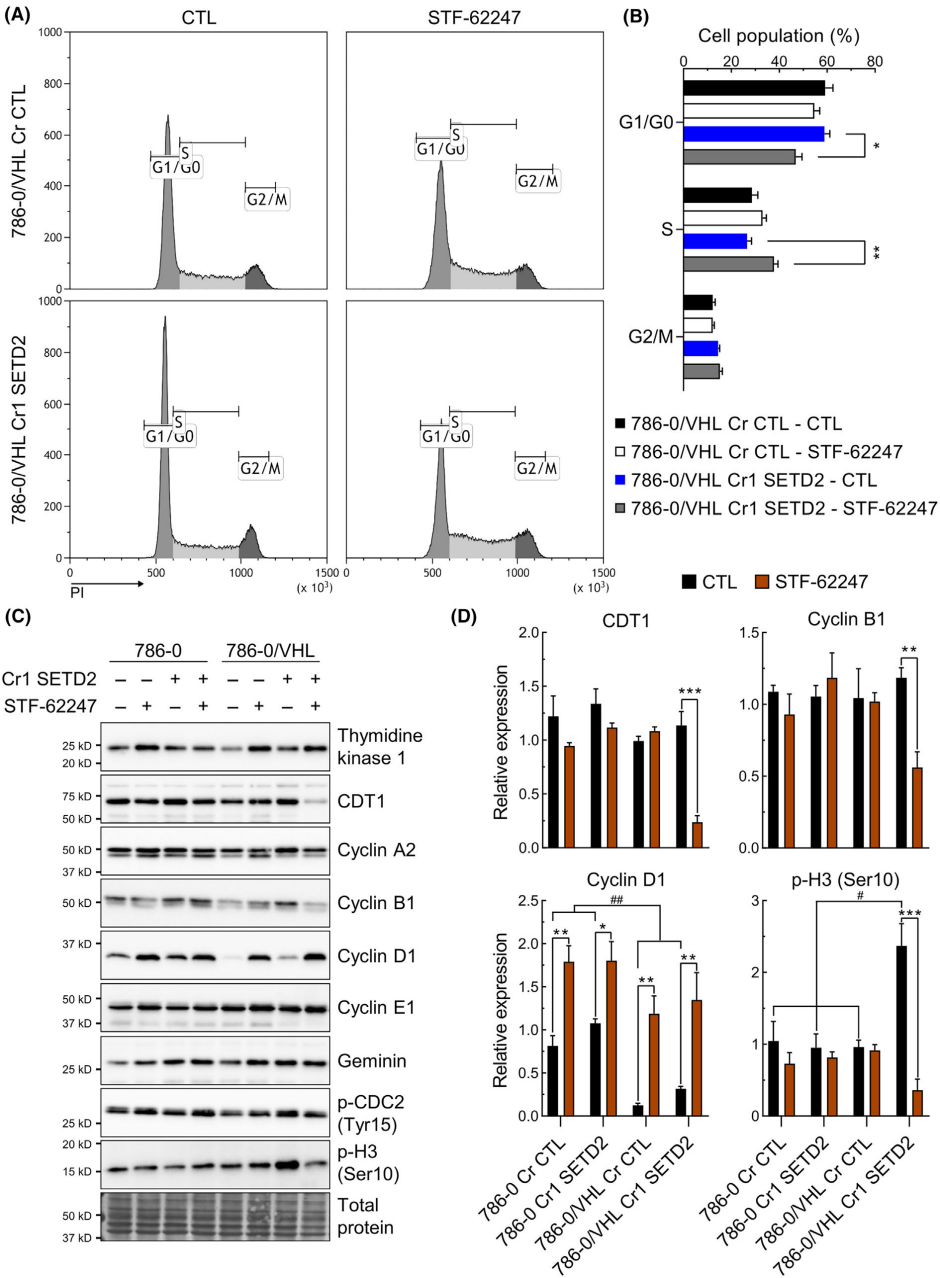
Decreased proliferation linked to cell cycle arrest in S‐phase. (A) Cell cycle analysis was performed by flow cytometry. 786‐0/VHL cells (Cr CTL and Cr1 SETD2) were treated with 3 μm STF‐62247 for 48 h before being fixed and stained with PI. (B) Cells were separated between cell cycle phases and represented by populations based on which phases they are found in. Results are presented as the mean ± SEM (*N* = 4). Statistically significant differences were tested with a two‐way ANOVA followed by Sidak's multiple comparisons test to compare CTL and STF‐62247 (*N* = 4). (C) Western blot was used to measure the expression of proteins implicated in the regulation of the cell cycle. (D) Quantification of western blot results in C. Results are presented as the mean ± SEM (*N* = 3). Statistically significant differences were tested with a two‐way ANOVA followed by Sidak's multiple comparisons test to compare CTL and STF‐62247 or Tukey's multiple comparisons test to compare between cell models (*N* = 3, Between CTL and STF‐62247 *P < 0.05, **P < 0.01, ***P < 0.001 or between cell models ^#^ *P* < 0.05, ^##^ *P* < 0.01).

Following the decrease in proliferation observed in STF‐62247‐treated cells, we used flow cytometry to analyze the cell cycle. Propidium iodide (PI) staining revealed a decrease in phase G1/G0 accompanied by an increase in S phase in 786‐0/VHL Cr1 SETD2 treated to STF‐62247 (Fig. 5A,B). Furthermore, we used antibodies targeting proteins relevant to each phase to measure their expression levels by western blot (Fig. 5C,D). In 786‐0/VHL Cr1 SETD2 treated with STF‐62247, there was a significant decrease of 78.2% of chromatin licensing and DNA replication factor 1 (CDT1) expression, a subunit of the pre‐replication complex, which is typically degraded in S phase. Additionally, cyclin B1 and phospho‐H3 (Ser10), both important in G2/M phases, were also decreased. These results indicate that STF‐62247 causes an arrest in S phase in 786‐0/VHL Cr1 SETD2, which would block the progression to G2/M. Cyclin D1 levels were significantly lower in *VHL*‐positive cells as expected since it is a target of HIF‐2α in ccRCC [42, 43]. Surprisingly, cyclin D1, which is often overexpressed in cancers and considered as an oncogene, was increased in all models treated with STF‐62247. Even though there was no significant indication of cell cycle arrest, other than for the 786‐0/VHL Cr SETD2, decreased proliferation was observed in all models and could influence cyclins levels. Additionally, Cyclin D1 can be degraded by selective autophagy, and by blocking autophagy with STF‐62247, Cyclin D1 could potentially accumulate in the cells [44]. For the 786‐0/VHL Cr1 SETD2 GFP‐SETD2, cyclin B1 is the only protein that behaved as it did in 786‐0/VHL Cr CTL cells, which is unaffected by STF‐62247 compared with the decrease seen in 786‐0/VHL Cr1 SETD2 (EV) (Fig. S7A,B).

### 3.6 DNA damage and loss of CDC25A are linked to S phase arrest

**Fig. 6:**
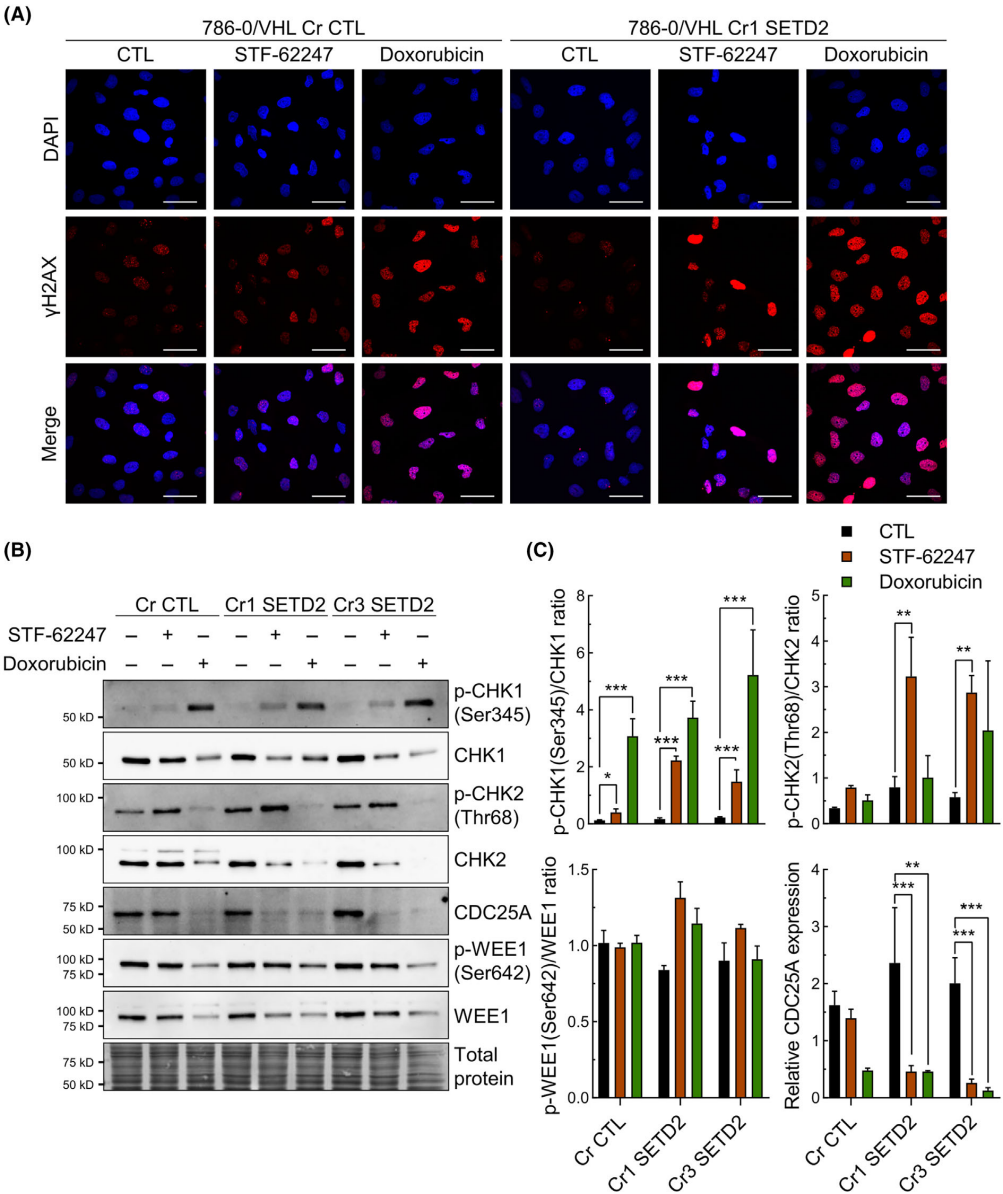
DNA damage leads to the loss of CDC25A. (A) DNA damage was measured by confocal microscopy using an antibody targeting γH2AX (red). DAPI was used to stain nuclei (blue). Cells (786‐0/VHL Cr CTL and Cr1 SETD2) were treated with 3 μm STF‐62247 for 48 h. As a positive control, cells were treated with 1 μm doxorubicin for 4 h (Scale bar = 50 μm). (B) Western blot analysis of CHK1, CHK2, p‐CHK1, p‐CHK2, CDC25A, WEE1, and p‐WEE1 in 786‐0/VHL Cr CTL, Cr1 SETD2 and Cr3 SETD2 treated with 3 μm STF‐62247 for 48 h. As a positive control, cells were treated with 0.5 μm doxorubicin for 24 h. (C) Quantification of western blot results obtained in C. Results are presented as the mean ± SEM (*N* = 3). Statistically significant differences were tested with a two‐way ANOVA followed by Dunnett's multiple comparisons test to compare each treatment with the control (*N* = 3, *P < 0.05, **P < 0.01 ***P < 0.001).

To better understand the cause behind that cell cycle arrest in the S phase, we explored any indications of DNA damage. Our results indicated a prominent increase in γH2AX, assessed by immunofluorescence, in 786‐0/VHL Cr1 SETD2 exposed to STF‐62247, suggesting the presence of DNA damage (Fig. 6A). Doxorubicin was used as a positive control. Then, phosphorylation ratios of checkpoint kinase 1 and 2 (CHK1 and CHK2) were increased in the two clones of Cr SETD2 when treated to STF‐62247, although these results were mostly due to the decrease in CHK1 and CHK2 expressions (Fig. 6B,C). The M‐phase inducer phosphatase 1 (CDC25A) implicated in cell cycle progression from phase S to G2 can be targeted by both CHK1 and CHK2 for degradation [45, 46]. Thus, we measured an important decrease of 81.5% in CDC25A protein expression in Cr1 SETD2 and 87.0% in Cr3 SETD2 treated with STF‐62247, coinciding with cell cycle arrest. No significant changes were observed on WEE1, the kinase responsible for the antagonist activity of CDC25A. These results indicate an important loss of CDC25A in *SETD2*‐knockout cells, most likely by degradation due to DNA damage, reinforcing evidence of S phase arrest.

### 3.7 Characteristics of pyroptosis in *SETD2* ‐knockout cells treated with STF‐62247

**Fig. 7:**
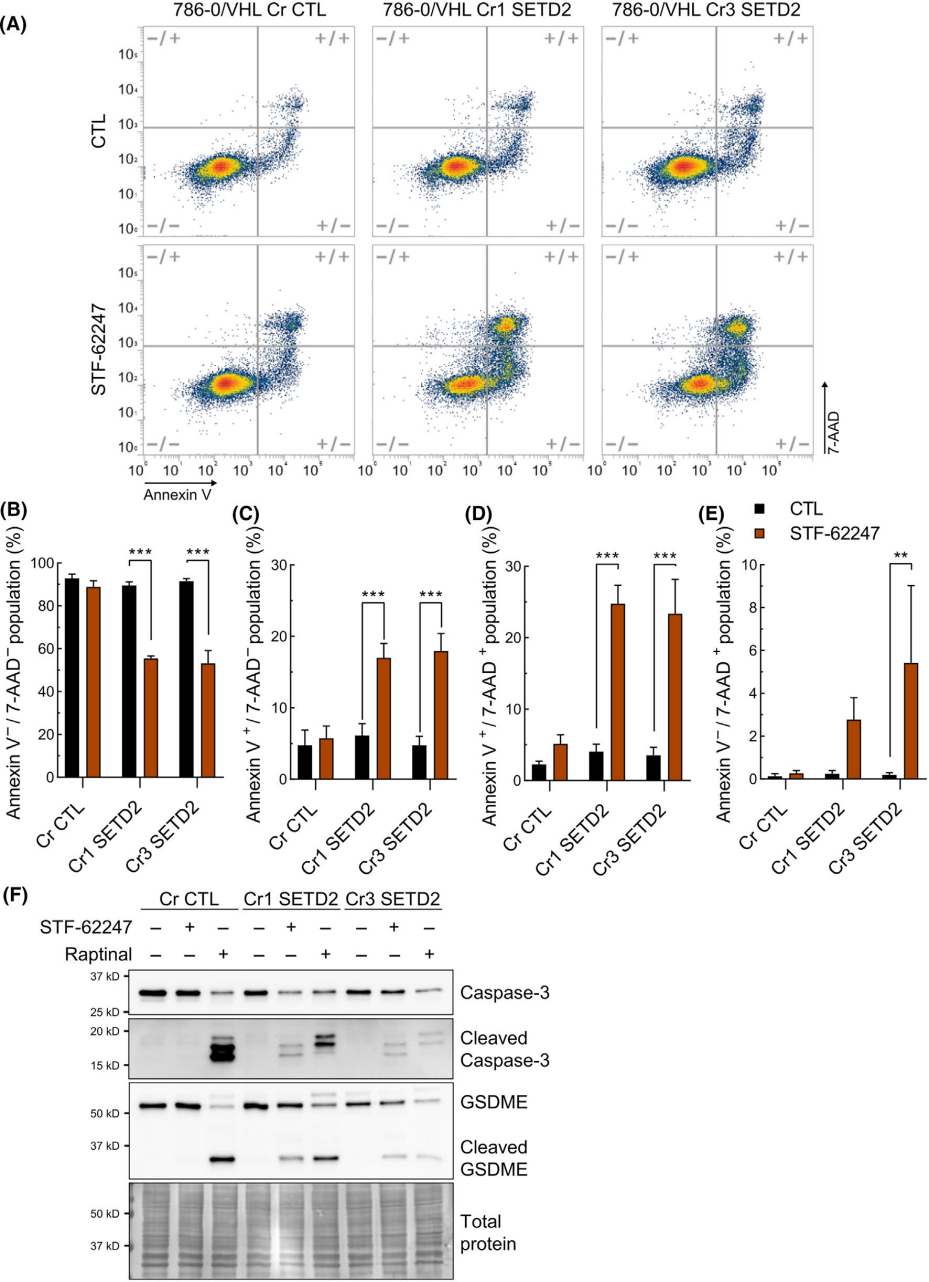
STF‐62247 treatment leads to pyroptosis‐like cell death in *SETD2*‐inactivated cells. (A) Apoptosis and cell death was measured by flow cytometry. 786‐0/VHL cells (Cr CTL, Cr1 SETD2, and Cr3 SETD2) were treated with 3 μm STF‐62247 for 48 h and incubated with annexin V and 7‐AAD before data acquisition. The following cell populations, based on annexin V and 7‐AAD staining, were obtained: (B) Annexin V^−^/7‐AAD^−^, (C) Annexin V^+^/7‐AAD negative, (D) Annexin V^+^/7‐AAD+, and (E) Annexin V^−^/7‐AAD^+^. Statistically significant differences between CTL and STF‐62247 was measured by two‐way ANOVA followed by Sidak's multiple comparisons test. Results are presented as the mean ± SEM (*N* = 3, **P < 0.01, ***P < 0.001). (F) Cleavage of caspase‐3 and GSDME was measured by western blot analysis. 786‐0/VHL cells (Cr CTL, Cr1 SETD2, and Cr3 SETD2) were treated with 3 μm STF‐62247 for 48 h or, as a positive control, with 10 μm raptinal for 2 h (*N* = 3).

Considering our results and the interconnection between DNA damage and apoptosis, we stained cells with annexin V and 7‐AAD to measure apoptosis and cell death by flow cytometry (Fig. 7A). When 786‐0/VHL Cr CTL cells were treated with STF‐62247, there was no significant difference in annexin V and 7‐aminoactinomycin D (7‐AAD) staining. However, in cells expressing SETD2 Cr1 and Cr3, we found a decrease in viable cells (Annexin V^−^/7‐AAD^−^) going from 89.6% to 55.4% and 91.5% to 53.2%, respectively (Fig. 7B). This reduction coincided with a significant increase in cells that are positively stained to only annexin V (Annexin V^+^/7‐AAD^−^) or to both markers (Annexin V^+^/7‐AAD^+^) (Fig. 7C,D). Only a small percentage of the population in Cr1 and Cr3 SETD2 treated with STF‐62247 was solely marked with 7‐AAD (Annexin V^−^/7‐AAD^+^) (Fig. 7E). These results suggest that, in addition to cell cycle arrest, 786‐0/VHL cells with *SETD2*‐depletion undergo apoptosis in response to STF‐62247.

To further assess the presence of apoptosis, we measured the cleavage of caspase‐3. Since it has been reported by others that cleaved caspase‐3 can lead to GSDME cleavage, a hallmark of cells undergoing pyroptosis, we studied these characteristics by western blot analysis [47]. We used raptinal, an activator of caspase‐3 and inducer of pyroptosis, as a positive control. As expected, we did not observe any cleavage of caspase‐3 or GSDME in 786‐0/VHL Cr CTL treated with STF‐62247. Notably, both caspase‐3 and GSDME were cleaved in the two 786‐0/VHL Cr SETD2 clones (Fig. 7F). These results were confirmed in RCC4/VHL (Fig. S8). Altogether, our results confirmed an annexin V positive cell death in 786‐0/VHL Cr SETD2, with the cleavage of caspase‐3 and GSDME suggesting that cells undergo pyroptosis.

## 4 Discussion

In the past two decades, we have seen major advancements in therapeutic approaches for ccRCC, especially with the development of targeted therapy and novel immunotherapies [48]. However, in many cases, primary or acquired resistance to therapies is still a challenge for treating patients with advanced kidney cancer. With new knowledge on ccRCC evolution linked to alterations of multiple genes such as *VHL*, *PBRM1*, *SETD2*, and *BAP1*, there is great potential to adopt new approaches anchored in the idea of precision medicine, either for monotherapy or combination therapy. The idea of using lysosomal disruption agents to improve cell response to treatments was particularly relevant with sunitinib, which can be sequestered in lysosomes and cause resistance [49, 50, 51]. Accordingly, we and others have highlighted the potential of using lysosomal‐disrupting agents or autophagy inhibitors as treatments for kidney cancer [21, 52, 53].

We previously characterized how STF‐62247 blocks late stages of autophagy in *VHL*‐deficient cells [22]. In the present study, we investigated how ccRCC mutational signature could affect cell responses to STF‐62247. Our results clearly indicated that *SETD2*‐knockout cells expressing WT‐*VHL* are sensitive to STF‐62247 and PIKfyve inhibitors. Although uncommon, ccRCC expressing WT‐*VHL* with mutations on *SETD2* are found in The Cancer Genome Atlas (TCGA) cohort for Kidney Renal Clear Cell Carcinoma (KIRC) as well as in the TRACERx Renal cohort [8, 54]. Notably, those cases from the latter cohort were all advanced cancers classified as stage III or IV. In addition to our previous work, we showed a tendency for VHL to protect cells from agents targeting autophagy and lysosomes but how inactivation of *SETD2* in *VHL*‐positive cells influences autophagy, precisely in response to STF‐62247, is still under investigation. However, in the past few years, a handful of studies have linked SETD2 to autophagy. SETD2 was first reported to play a role in autophagy by regulating the autophagy‐related (ATG) protein, ATG12 [55]. In cells lacking SETD2, a short isoform of ATG12 was produced due to alternative splicing causing an accumulation of free ATG12. Another group also studied the ATG5‐ATG12 complex and showed conflicting results with no significant changes in levels of free ATG12 when SETD2 was altered [56]. Although it is still unclear exactly how SETD2 regulates ATG12, this latest group also showed that SETD2 methylates actin and allows the regulation of the protein WASP homolog associated with actin, Golgi membranes and microtubules (WHAMM), which plays a role in lysosomal tubulation [15]. Finally, the protein FIP200, implicated in autophagosome formation, is one of the latest targets found to be methylated by SETD2 [12]. By methylating FIP200 on residue lysine 1133, SETD2 blocks its recognition by the E3 ligase tripartite motif containing 21 (TRIM21), thus preventing its ubiquitination and degradation.

Consistent with the era of precision medicine, there is a growing interest in studying *SETD2* as a tumor suppressor gene and finding ways to selectively target *SETD2*‐deficient cancer cells. One of the first targeted proteins identified was PI3Kβ, whose inhibition by TGX221 or AZD6482 was more lethal when SETD2 was missing [57]. We were able to replicate these results with our *SETD2*‐inactivated cells being more sensitive to AZD8186. Since PI3Kβ and PIKfyve are both kinases related to phosphoinositides, the interrelation between SETD2 and phosphoinositides metabolism would be of interest to move forward as, to our knowledge, this field has not been studied.

By assessing the impact of SETD2 on cell responses to STF‐62447, we not only demonstrated complete inhibition of proliferation in *SETD2*‐knockout cells after treatment with STF‐62247, but we also showed that the cells were blocked in S phase, corroborated by a decrease of CDT1. This is particularly interesting because SETD2 was reported to play a role in the response to DNA replication stress. When cells are exposed to conditions causing replication stress, SETD2 presence on chromatin increases, allowing trimethylation of histone 3 on lysine 14 (H3K14me3) [58]. This information adds up to the already well‐known implication of SETD2 in DNA damage repair through the regulation of H3K36me3. After the formation of DNA double‐strand breaks, H3K36me3 marks accumulate rapidly and are required for the consequent recruitment of PC4 and SF2 interacting protein (LEDGF) and lysine acetyltransferase 5 (KAT5) for the acetylation of H4K16 (H4K16ac) and subsequent modulation of chromatin openness and recruitment of DNA repair proteins [59].

Our results showed an increase in CHK1 and CHK2 phosphorylation ratios indicating their activation, but we noticed that their protein levels were lower with STF‐62247 or doxorubicin. This phenomenon has been previously reported in RCC4/VHL treated with DNA damage‐inducing agents where the increase in CHK1 phosphorylation on S345 was accompanied by an important decrease in CHK1 protein levels [60]. It was also recently reported that *SETD2*‐inactivation sensitizes cells to ataxia telangiectasia and Rad3‐related protein (ATR) inhibition [61]. Inhibition of either ATR or CHK1 in *SETD2*‐inactivated cells caused an accumulation of cytosolic DNA, thus activating the cyclic GMP‐AMP synthase‐stimulator of interferon genes (cGAS‐STING) pathway and the subsequent production of cytokines, including interferon‐alpha (IFNα). Thus, inactivation of *SETD2* and inhibition of ATR stimulate immune cell infiltration in the microenvironment and enhance the response to immunotherapy. This was demonstrated by cotreatment with a PD‐1 antibody and an ATR inhibitor, which significantly decreased tumor growth in mice subcutaneously injected with *SETD2*‐inactivated Renca cells [61]. Other groups have also shown a better response to immune checkpoint inhibitors in different cancers deficient in *SETD2* [62, 63].

To follow up on the indications of DNA damage, we showed an almost complete loss of CDC25A, specifically in *SETD2*‐knockout cells treated with STF‐62247. This is particularly relevant because *SETD2*‐deficient cells showed promising results for the WEE1 inhibitor, AZD1775 (Adavosertib), which went to phase II clinical trial for *SETD2*‐deficient solid tumors, including ccRCC [64]. In fact, it was first reported that ccRCC cells with mutations on *SETD2* were vulnerable to AZD1775 and treatments stopped tumor growth *in vivo* [65]. Unfortunately, results from the trial failed to show objective responses; however, there was a prolonged period of stable disease in more than half of the cohort. Also linked to WEE1, it has been shown that polo‐like kinase 1 (PLK1) was more expressed in ccRCC deficient in *SETD2* [66]. This was not observed in cases expressing WT‐VHL due to the regulation of PLK1 expression by HIF‐2α. Additionally, PLK1 inhibitors have been shown to induce GSDME‐dependent pyroptosis in colorectal and ovarian cancers [67, 68]. Regarding pyroptosis, cleavage of caspase‐3 and GSDME was detected in *SETD2*‐knockout cells expressing *VHL* following treatment with STF‐62247. Similarly, *SETD2* depletion was recently shown to confer vulnerability to poly(ADP‐ribose) polymerase 1 (PARP) and DNA methylation dual inhibition, leading to the cleavage of caspase‐3 [69]. Interestingly, a similar drug combination leads to pyroptosis in prostate cancer [70].

Overall, results showing vulnerability to STF‐62247 and PIKfyve inhibitors in WT‐*VHL* ccRCC cells with *SETD2*‐loss are intriguing, and part of the answer could come from the disruption in the autophagic flux by VHL and SETD2. As previously reported, *SETD2*‐loss was associated with the downregulation of autophagy whether through the deregulation of ATG12, WHAMM, or FIP200 [12, 55, 56]. Meanwhile, VHL can target and ubiquitinate both Beclin1 and MAP1LC3B, leading to their degradation and inhibition of autophagy initiation [71, 72]. Both VHL and SETD2 also play a role in stabilizing microtubules, VHL by binding to α‐tubulin and preventing depolymerization, and SETD2 by methylating α‐tubulin for genomic stability [73, 74]. This is particularly relevant for lysosome motility on microtubules. We previously demonstrated that *VHL* status influenced lysosome localization during STF‐62247 treatments, but the repercussion of microtubule destabilization on lysosome motility in *SETD2*‐deficient cells is still unknown [27]. But the question remains: Why are *VHL*‐positive cancer cells, other than ccRCC, not vulnerable to STF‐62247 and PIKfyve inhibitors after *SETD2*‐inactivation? Almost all ccRCCs share a common evolutionary path involving the initial loss of chromosome 3p that can lead to haploinsufficiency of some genes and impact cell response to drugs, *SETD2* being a great example [74]. Finally, variations in cellular pathways could influence how cancer cells respond to PIKfyve inhibitors. For instance, it has been shown that targeting both PIKfyve and tubulin or combined inhibition of PIKfyve and p38MAPK can target certain cancer cells [75, 76]. Moreover, a group demonstrated that cells with low levels of PIP5K1C are more vulnerable to PIKfyve inhibitors [77]. Therefore, it is clear that specific characteristics acquired by cancer cells can dictate their response to drugs, which highlights the intricacy behind choosing cancer treatments.

## 5 Conclusion

To summarize, while we have shown that loss of *VHL* or *SETD2* could significantly improve the response to PIKfyve inhibitors, it is also evident that there is much knowledge that is yet to be discovered on the interconnection between mutated genes in ccRCCs and how it may influence therapeutic responses. Considering the documented impact of the loss of *SETD2* on the microenvironment and how pyroptosis leads to the extracellular release of cytokines, combination of STF‐62247 and immune checkpoint inhibitors would be an interesting avenue to investigate further to assess whether a combination of these two treatments could be beneficial. Nevertheless, we demonstrated the potential of STF‐62247 and other PIKfyve inhibitors to target the aggressive WT‐*VHL* ccRCC subtype in cases where *SETD2* is inactivated.

## Conflict of interest

The authors declare no conflict of interest.

## Author contributions

MJ and ST conceived and designed the research. MJ performed the experiments, analyzed the data, interpreted the results, and prepared the figures under the supervision of ST. MJ wrote the manuscript. ST revised and approved the final version of the manuscript.

### Peer review

The peer review history for this article is available at https://www.webofscience.com/api/gateway/wos/peer‐review/10.1002/1878‐0261.13770.

## Supporting information


**Fig. S1.** Additional results of XTT assay in ccRCC cell lines.
**Fig. S2.** Sensitivity to STF‐62247 and apilimod confirmed in additional clone of 786‐0/VHL Cr SETD2.
**Fig. S3.** Vulnerability to STF‐62247 and PIKfyve inhibitors in RCC4/VHL Cr SETD2 cells.
**Fig. S4.** Non‐ccRCC cell lines responses to vacuolin‐1 and APY0201.
**Fig. S5.** Decrease in proliferation and viability in additional clone of 786‐0/VHL Cr SETD2 treated with STF‐62247.
**Fig. S6.** Decrease in proliferation and viability confirmed in RCC4/VHL Cr SETD2 clones.
**Fig. S7.** Cell cycle‐related proteins in GFP‐SETD2 model.
**Fig. S8.** Pyroptosis‐like cell death observed in RCC4/VHL Cr SETD2 cells.
**Table S1.** Culture medium used for the cell lines.
**Table S2.** List of drugs used in this study.
**Table S3.** List of antibodies used in this study.
**Table S4.** List of primers for CRISPR/Cas9 gene editing.

## Data Availability

The data that support the findings of this study are available from the corresponding author upon reasonable request.
