# Supplementary material for: Loss of SETD2 in wild‐type VHL clear cell renal cell carcinoma sensitizes cells to STF‐62247 and leads to DNA damage, cell cycle arrest, and cell death characteristic of pyroptosis
Source: Mol Oncol. 2024 Nov 26;19(4):1244–64. doi: 10.1002/1878-0261.13770 (PMC11977649; doi:10.1002/1878-0261.13770)
Supplement: Supplementary file 1 — Fig. S1. Additional results of XTT assay in ccRCC cell lines. Fig. S2. Sensitivity to STF‐62247 and apilimod confirmed in additional clone of 786‐0/VHL Cr SETD2. Fig. S3. Vulnerability to STF‐62247 and PIKfyve inhibitors in RCC4/VHL Cr SETD2 cells. Fig. S4. Non‐ccRCC cell lines responses to vacuolin‐1 and APY0201. Fig. S5. Decrease in proliferation and viability in additional clone of 786‐0/VHL Cr SETD2 treated with STF‐62247. Fig. S6. Decrease in proliferation and viability confirmed in RCC4/VHL Cr SETD2 clones. Fig. S7. Cell cycle‐related proteins in GFP‐SETD2 model. Fig. S8. Pyroptosis‐like cell death observed in RCC4/VHL Cr SETD2 cells. Table S1. Culture medium used for the cell lines. Table S2. List of drugs used in this study. Table S3. List of antibodies used in this study. Table S4. List of primers for CRISPR/Cas9 gene editing. [file MOL2-19-1244-s001.pdf]

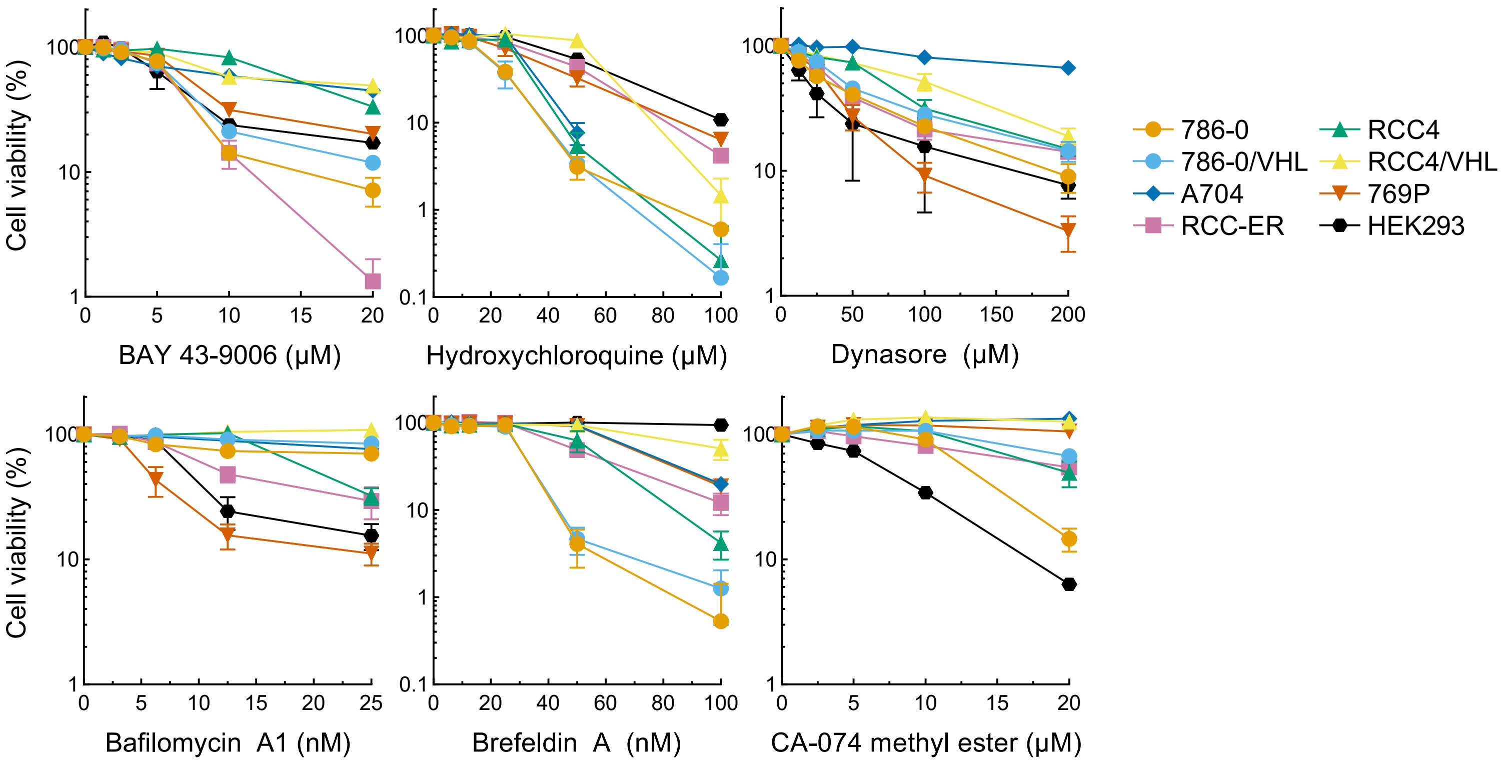

Figure S1. Additional results of XTT assay in ccRCC cell lines. Cell viability in ccRCC cell lines measured by XTT assay. Cells were treated with a variety of drugs for 72 h. Results are presented as the mean ± SEM (N=3).

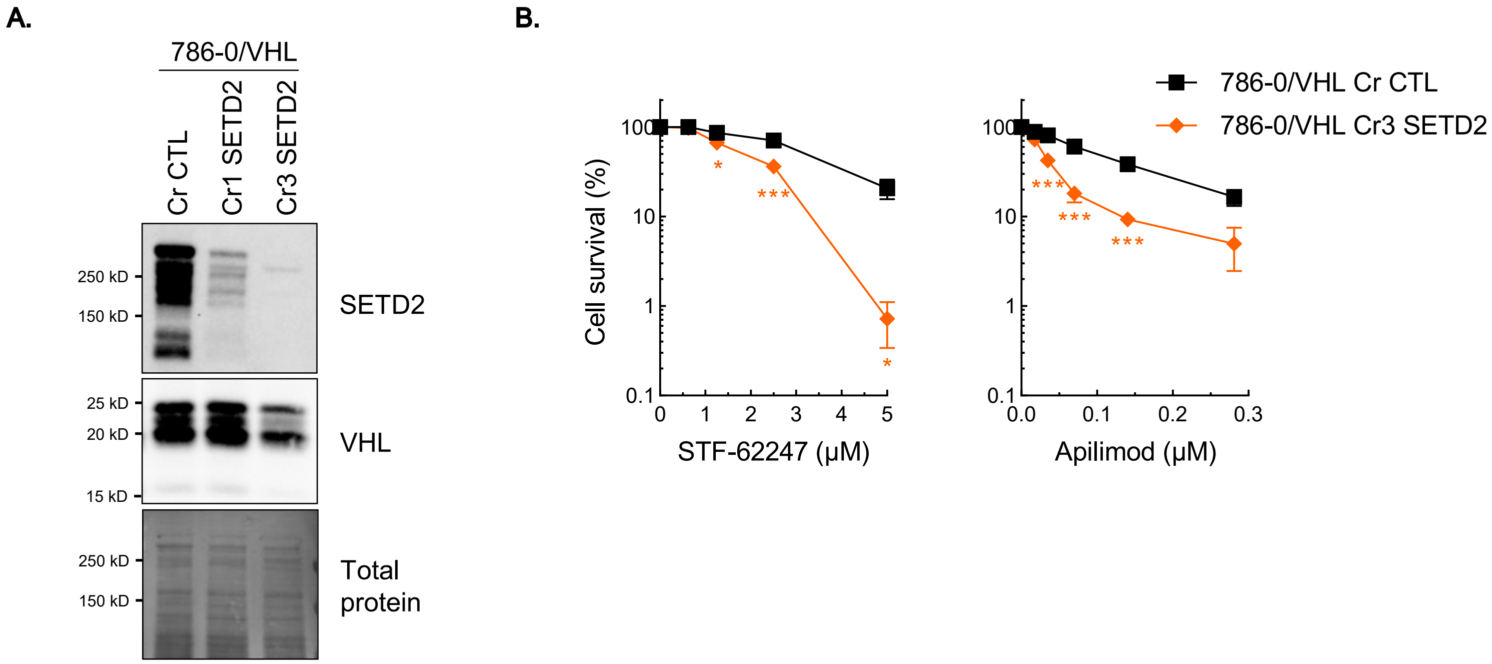

Figure S2. Sensitivity to STF-62247 and apilimod confirmed in additional clone of 786-0/VHL Cr SETD2. A) Validation of clone 786-0/VHL Cr3 SETD2 by western blot. **B)** Cell survival of 786-0/VHL Cr3 SETD2 measured by clonogenic assay with treatments to STF-62247 or apilimod. Results are compared to 786-0/VHL Cr CTL (same as Figure 2B). Statistically significant differences between Cr CTL and Cr SETD2 were tested with two-way ANOVA followed by Sidak's multiple comparisons test. Results are presented as the mean ± SEM (N=3, *$p<0.05$, ***$p<0.001$)

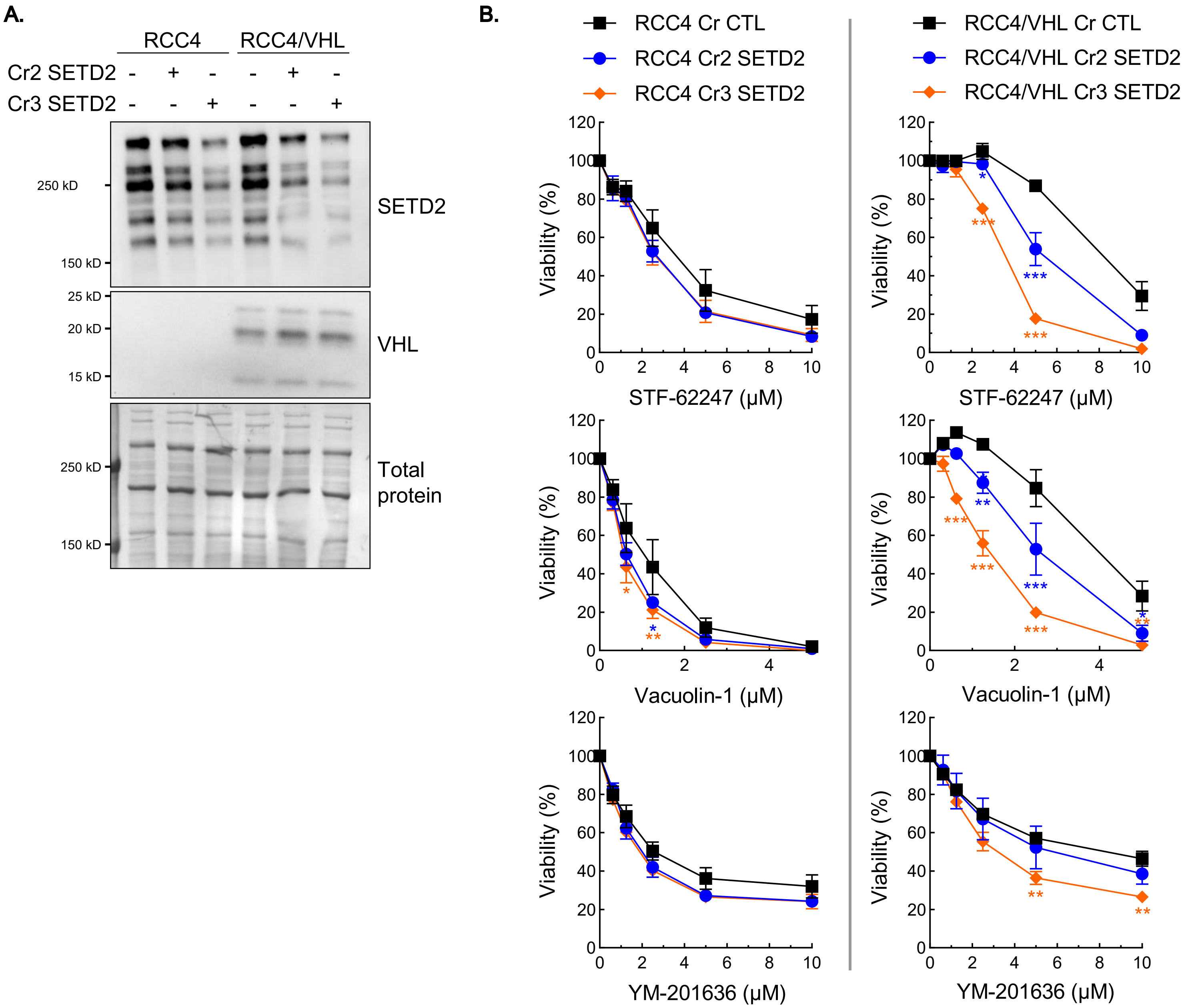

Figure S3. Vulnerability to STF-62247 and PIKfyve inhibitors in RCC4/VHL Cr SETD2 cells. A) Western blot to validate gene repression of SETD2 by CRISPR/Cas9 in RCC4 and RCC4/VHL cells. **B)** Cell viability was measured by XTT assay with treatments of STF-62247 and PIKfyve inhibitors. RCC4/VHL Cr SETD2 acquired a clear vulnerability to STF-62247 and Vacuolin-1. Statistical significance was measured using two-way ANOVA followed by Dunnett's multiple comparisons test to compare Cr2 SETD2 or Cr3 SETD2 with Cr CTL. Results are presented as the mean ± SEM (N=3, *p<0.05, **p<0.01, ***p<0.001)

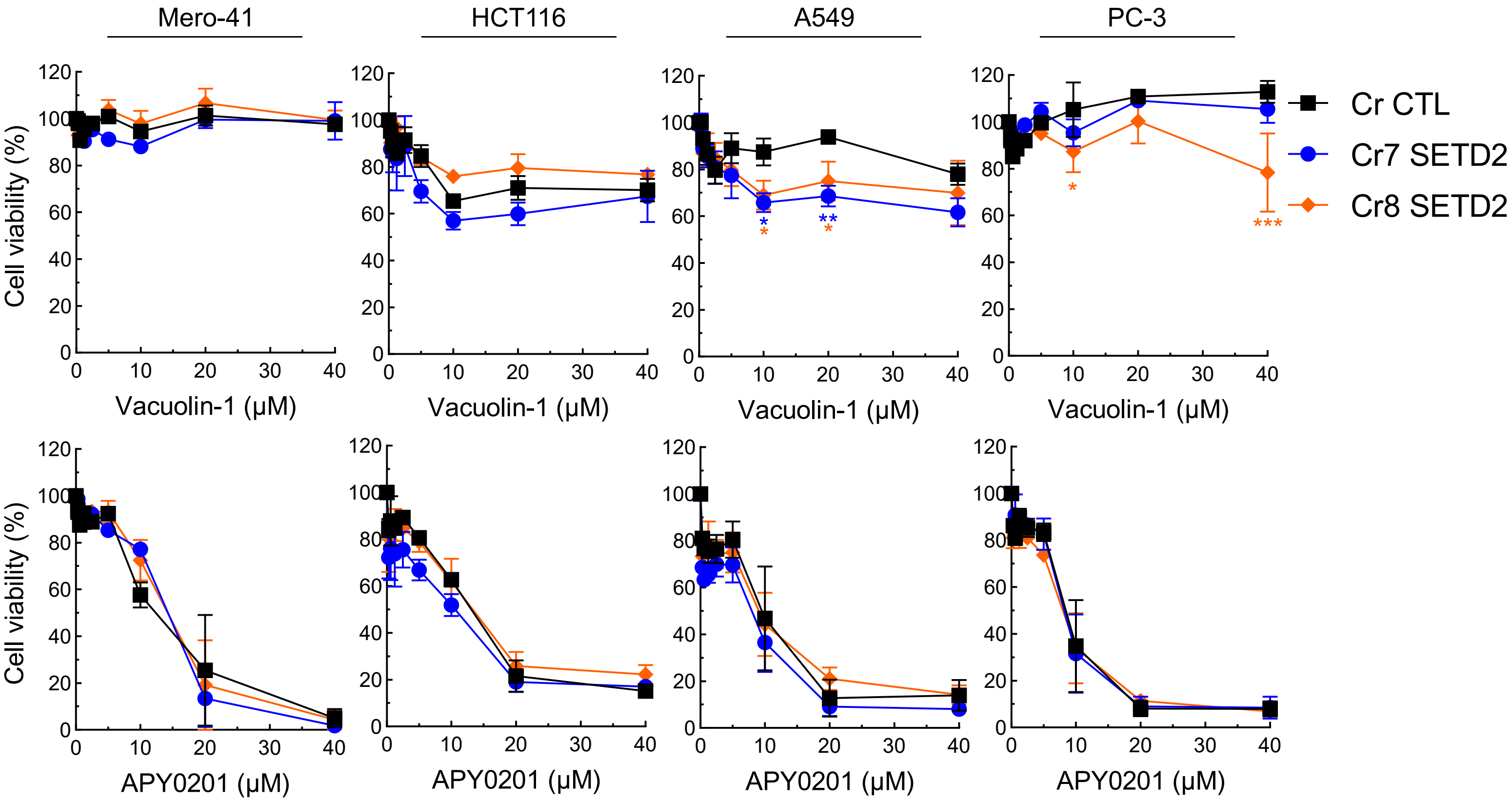

Figure S4. Non-ccRCC cell lines responses to vacuolin-1 and APY0201. Cell viability was measured by XTT assay in Mero-41 (mesothelioma), HCT116 (colon cancer), A549 (lung cancer) and PC-3 (prostate cancer) cells, with and without SETD2. Cells were treated with PIKfyve inhibitors (Vacuolin-1 and APY0201) for 72 h. Statistical significance was measured using two-way ANOVA followed by Dunnett's multiple comparisons test to compare Cr7 SETD2 or Cr8 SETD2 with Cr CTL. Results are presented as the mean ± SEM (N=3, *p<0.05, **p<0.01, ***p<0.001)

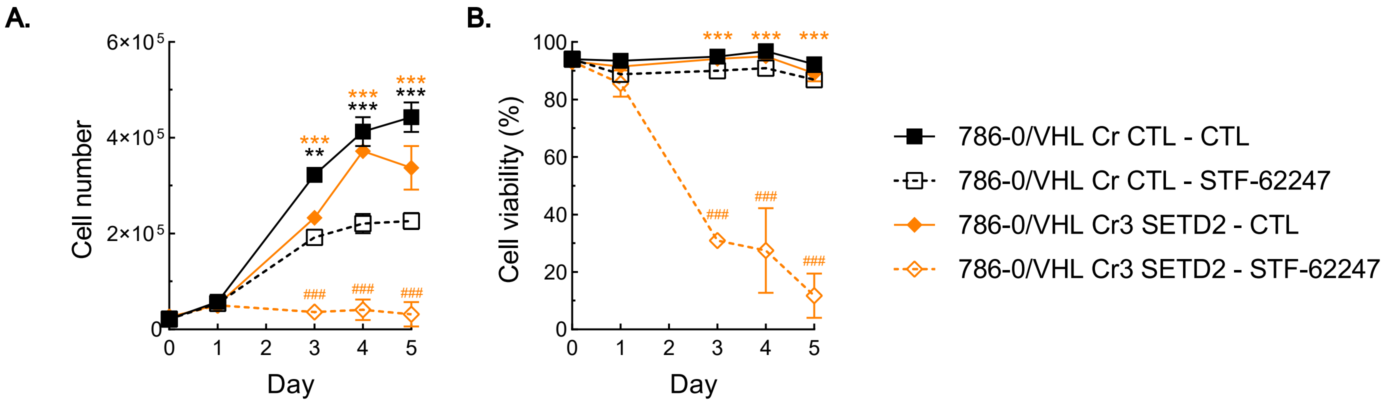

Figure S5. Decrease of proliferation and viability in additional clone of 786-0/VHL Cr SETD2 treated with STF-62247. A) Proliferation and **B)** cell viability measured by cell counts with trypan blue. 786-0/VHL Cr3 SETD2 clone was treated with 3 µM STF-62247 at day 0. Results are compared to 786-0/VHL Cr CTL (same as Figure 4A-B). Results are presented as the mean ± SEM (N=3). Statistically significant differences were tested with a two-way ANOVA followed by Tukey's multiple comparisons test. Comparisons between CTL and STF-62247 are indicated with the * symbol and comparisons between Cr3 SETD2 and Cr CTL are indicated with the # symbol.

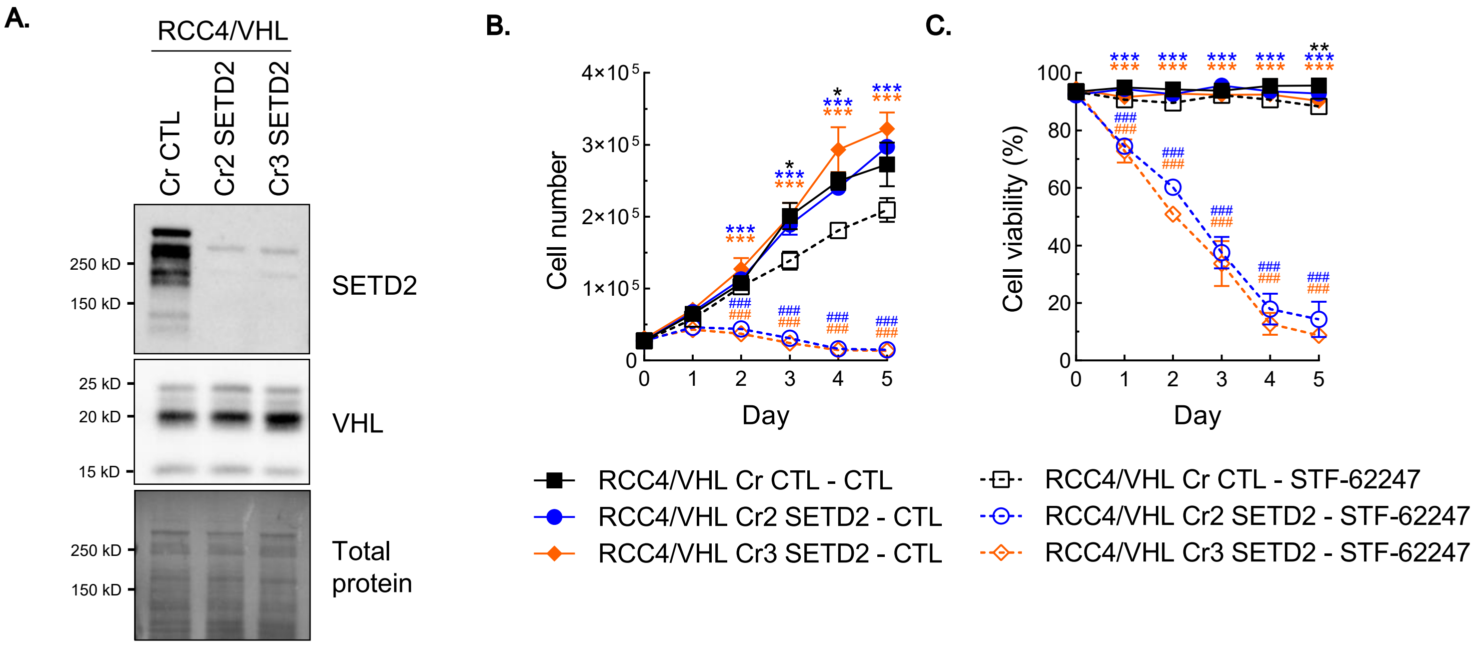

Figure S6. Decrease of proliferation and viability confirmed in RCC4/VHL Cr SETD2 clones. A) Preparation and validation, by western blot, of clones in RCC4/VHL Cr2 and Cr3 SETD2. **B)** Proliferation and **C)** cell viability measured by cell counts with trypan blue. Cells were treated with 1.5 µM STF-62247 at day 0. Results are presented as the mean ± SEM (N=3). Statistically significant differences were tested with a two-way ANOVA followed by Tukey's multiple comparisons test. Comparison between CTL and STF-62247 are indicated with the * symbol and comparisons between Cr 2 or Cr3 SETD2 and Cr CTL are indicated with the # symbol. (*p<0.05, **p<0.01, ***p<0.001 or ###p<0.001).

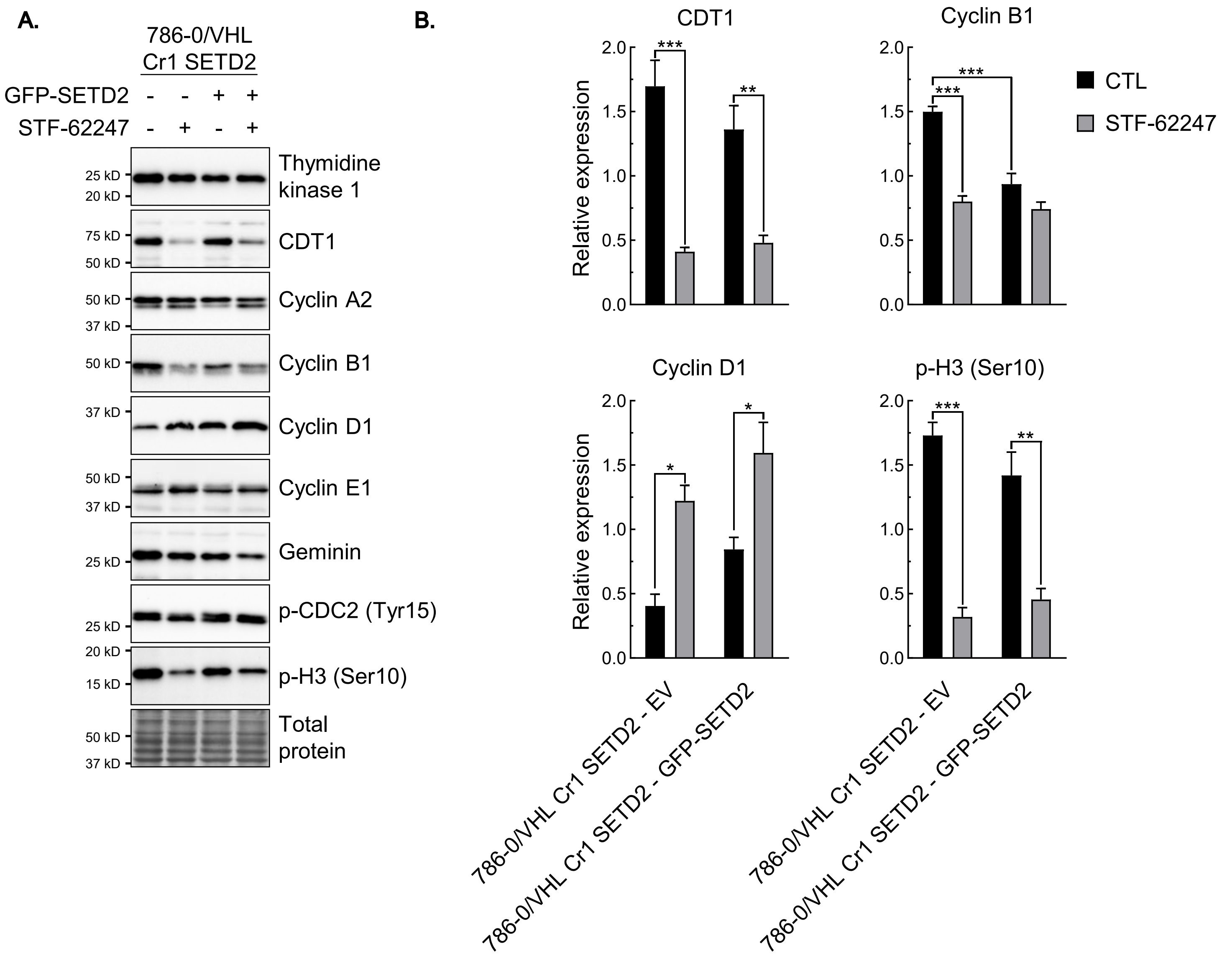

Figure S7. Cell cycle-related proteins in GFP-SETD2 model. A) Western blot analysis of cell cycle phases in 786-0/VHL Cr1 SETD2 (EV) and 786-0/VHL Cr1 SETD2 + GFP-SETD2 treated with 3 μM STF-62247 for 48 h. **B)** Quantification of the proteins impacted by STF-62247 in 786-0/VHL Cr 1 SETD2. Statistically significant differences were tested with a two-way ANOVA followed by Sidak's multiple comparisons test. Results are presented as the mean ± SEM (N=3, *p<0.05, **p<0.01, ***p<0.001).

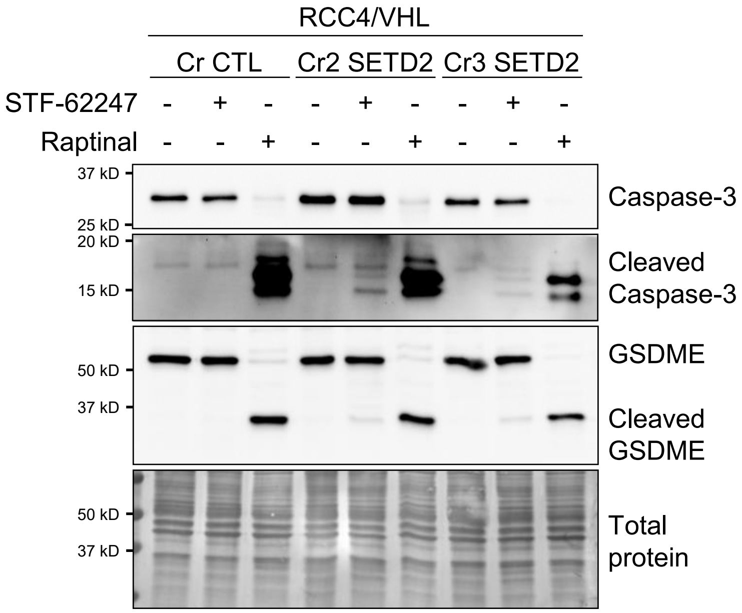

Figure S8. Pyroptosis-like cell death observed in RCC4/VHL Cr SETD2 cells. Western blot analysis confirms that caspase-3 and gasdermin E are cleaved in RCC4/VHL Cr SETD2 treated with STF-62247. Cells were treated with 1.5 µM STF-62247 for 48 h or 10 µM raptinal for 2 h.

Table S1. Culture medium used for the cell lines

Cell line	Media	Supplements
786-0	DMEM (Wisent, 319-030-CL)	10% FBS (Wisent, 080-150) + 2 mM Gln (Wisent, 609-065-EL) + 1 mM Sodium pyruvate (HyClone, SH30239.01)
RCC4	DMEM	10% FBS + 2 mM Gln + 1mM Sodium pyruvate
A704	EMEM (Wisent, 320-005-CL)	10% FBS + 1 mM Sodium pyruvate + 1% Non-Essential Amino Acids
RCC-ER	RPMI 1640 (Wisent, 350-000-CL)	10% FBS
769P	RPMI 1640	10% FBS + 1 mM Sodium pyruvate
HEK293	EMEM	10% FBS + 1 mM Sodium pyruvate + 1% Non-Essential Amino Acids
Mero-41	Ham's F-10 (Wisent, 318-051-CL)	15% FBS
A549	DMEM	10% FBS + 2 mM Gln
HCT116	McCoy's (Wisent, 317-010-CL)	10% FBS
PC-3	RPMI 1640	10% FBS

Table S2. List of drugs used in this study

Reagent	Manufacturer	Catalog number
Apilimod	Selleckchem	S6414
APY0201	Cayman Chemical	9001589
Axitinib	Cayman Chemical	13813
AZD8186	Cayman Chemical	17384
Bafilomycin A1	Cayman Chemical	11038
BAY 43-9006	Cayman Chemical	10009644
Brefeldin A	Cayman Chemical	11861
CA-074 methyl ester	Cayman Chemical	18469
Cisplatin	Cayman Chemical	13119
Dynasore	Sigma Aldrich	D7693
Everolimus	Cayman Chemical	11597
Hydroxychloroquine	Cayman Chemical	30709
Pazopanib	Cayman Chemical	12097
Raptinal	AdipoGen	AG-CR1-2902-M001
Sunitinib	Cayman Chemical	13159
STF-62247	APExBIO	B2151
Vacuolin-1	Cayman Chemical	20425
YM-201636	Cayman Chemical	13576

Table S3. List of antibodies used in this study

Antibody	Host	Manufacturer	Catalog number
VHL	Rabbit	Cell Signaling	68547
HIF-1a	Rabbit	Cell Signaling	14179
HIF-2a	Rabbit	Cell Signaling	59973
PBRM1/BAF180	Rabbit	Cell Signaling	91894
SETD2	Rabbit	Cell Signaling	80290
BAP1	Rabbit	Cell Signaling	13271
H3K36me3	Rabbit	Cell Signaling	4909
H3	Rabbit	Cell Signaling	4499
Thymidine Kinase 1	Rabbit	Cell Signaling	28755
CDT1	Rabbit	Cell Signaling	8064
Cyclin A2	Rabbit	Cell Signaling	91500
Cyclin B1	Rabbit	Cell Signaling	12231
Cyclin D1	Rabbit	Cell Signaling	55506
Cyclin E1	Rabbit	Cell Signaling	20808
Geminin	Rabbit	Cell Signaling	52508
Phospho-CDC2 (Tyr15)	Rabbit	Cell Signaling	4539
Phospho-H3 (Ser10)	Rabbit	Cell Signaling	53348
CHK1	Rabbit	Abcam	Ab40866
Phospho-CHK1 (Ser345)	Rabbit	Cell Signaling	2341
CHK2	Rabbit	Cell Signaling	2662
Phospho-CHK2 (Thr68)	Rabbit	Cell Signaling	2661
WEE1	Rabbit	Cell Signaling	13084
Phospho-WEE1 (Ser642)	Rabbit	Cell Signaling	4910
CDC25A	Mouse	Santa Cruz	SC-7389
Caspase-3	Rabbit	Cell Signaling	14220
Gasdermin E	Rabbit	Cell Signaling	84005
γH2AX	Rabbit	Cell Signaling	9718

Table S4. List of primers for CRISPR/Cas9 gene editing

gRNA	Primer	Sequence (5'-3')
Cr1 SETD2	Forward	CACCGGAGAGTGTTGTGGCTTGGGC
	Reverse	AAACGCCCAAGCCACAACACTCTCC
Cr2 SETD2	Forward	CACCGGTTGTGTATGATCGAACTCA
	Reverse	AAACTGAGTTCGATCATACACAACC
Cr3 SETD2	Forward	CACCGTGCGGATCAGCCAATTGCCG
	Reverse	AAACCGGCAATTGGCTGATCCGCAC
Cr7 SETD2	Forward	CACCGGGACTGTGAACGGACAACTG
	Reverse	AAACCAGTTGTCCGTTCACAGTCCC
Cr8 SETD2	Forward	CACCGGACTGTGAACGGACAACTG
	Reverse	AAACTCAGTTGTCCGTTCACAGTCC
Cr PBRM1	Forward	CACCGTCTTCTTCTCTTGGAACCCA
	Reverse	AAACTGGGTTCCAAGAGAAGAAGAC
